# Mechanistic Studies of the Proton-Coupled Electron Transfer Reactivity of a Cobalt Complex with a Proton-Responsive PNP Pincer-Type Ligand

**DOI:** 10.1021/acs.inorgchem.5c01792

**Published:** 2025-07-10

**Authors:** Jyotima Mukherjee, Nils Ostermann, Jan Pecak, Matthias Otte, Maren Podewitz, Inke Siewert

**Affiliations:** 1 9375 Georg-August-Universität Göttingen, Institut für Anorganische Chemie, Tammannstr. 4, Göttingen 37077, Germany; 2 Institute of Materials Chemistry, 27259 TU Wien, Getreidemarkt 9, Wien 1060, Austria; 3 Georg-August-Universität Göttingen, International Center for Advanced Studies of Energy Conversion, Tammannstr. 4, Göttingen 37077, Germany

## Abstract

Herein, we investigate the proton-coupled electron transfer (PCET) reactivity of a cobalt­(I) complex with a proton-responsive pyridin-4-ol PNP pincer-type ligand (HL^PNP^ = 2,6-bis­((bis-*tert*-butylphosphaneyl)-methyl)­pyridin-4-ol). The cobalt­(II) complexes [(L^PNP^)­Co^II^Cl], **1**, and [(L^PNP^)­Co^II^(MeCN)]^+^, **2** ^**+**^, with the deprotonated ligand and [(HL^PNP^)­Co^II^(MeCN)_2_]^2+^, **2H** ^**2+**^, with the protonated ligand, were synthesized and characterized. **2H** ^**2+**^ has a p*K*_a_ of 18 ± 1, and the reduction of **2H** ^**2+**^ appears at −1.08 V vs. FeCp_2_^+|0^ in MeCN. This leads to a bond dissociation free energy (BDFE) of the OH bond in [(HL^PNP^)­Co^I^(MeCN)]^+^, **2H** ^**+**^, of 52 kcal mol^–1^, which is supported by DFT calculations. The solution BDFE of **2H** ^**+**^ equals the BDFE of ^1^/_2_ H_2_, and indeed, **2H** ^**+**^ slowly loses dihydrogen. Kinetic analysis revealed a first-order rate law in **2H** ^**+**^ with a reaction rate constant *k* of 3.2 × 10^–4^ s^–1^ at 25 °C and a positive activation entropy Δ*S* ^‡^ of 9.4 ± 0.6 cal (Δ*H* ^‡^ = 24.3 ± 0.2 kcal mol^–1^) for H_2_ loss. Based on these kinetic results, H/D labeling studies, and DFT calculations, a unimolecular mechanism is proposed. However, H atom transfer from **2H** ^**+**^ to acceptors such as (2,2,6,6-tetramethylpiperidin-1-yl)­oxyl or 2,4,6-*tert*-butylphenoxide is very fast (*k*_2_ of 10^4^ s^–1^ M^–1^ for the reaction of **2H** ^**+**^ with TEMPO^•^) and H_2_ loss can be easily outcompeted.

## Graphical abstract

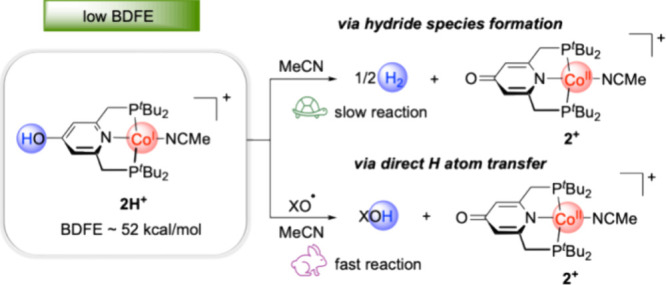

## Introduction

The renaissance of electro- or photoorganic methods for the synthesis of value-added organic compounds is driven by a recent interest in using renewable energy sources for chemical synthesis.^1^ The coupled transfer of electrons and protons is of fundamental importance in such electro- and photochemical conversions, e.g., hydrogenation reactions and C–H functionalization reactions.^2^ In order to reduce, e.g., C=C, C≡N bonds, by single H^+^/e^–^ transfer steps, reactants with low bond dissociation free energies (BDFEs) are desirable as the initially formed C–H bond is usually very weak.^3^ Metal hydrides have been explored as electroreduction catalysts because they can be tuned facilely and represent crucial intermediates in thermal hydrogenation reactions.^4^ However, metal hydride complexes with M–H BDFEs below 52 kcal mol^–1^ are also prone to H_2_ loss because ^1^/_2_ H_2_ has a solution BDFE of about 52 kcal mol^–1^, and thus, its formation is thermodynamically favored.^5^ In order to circumvent this drawback, metal complexes were recently explored, in which the proton and electron donor/acceptor sites are spatially separated because this could stabilize rather weak X–H bonds within the ligand framework.^6^ Due to the polarity mismatch of two “protic” H atoms, H_2_ loss could be decelerated. For example, this concept has been exploited for electrochemical synthesis in the cobaltocene-based redox mediator **I** depicted in Figure 1.^6g^ The complex has a very low BDFE of 39 kcal mol^–1^ and acts as a redox mediator in the electroreduction of various substrates, including C=C and C=O bonds.^6g,6h^

**Figure 1:**
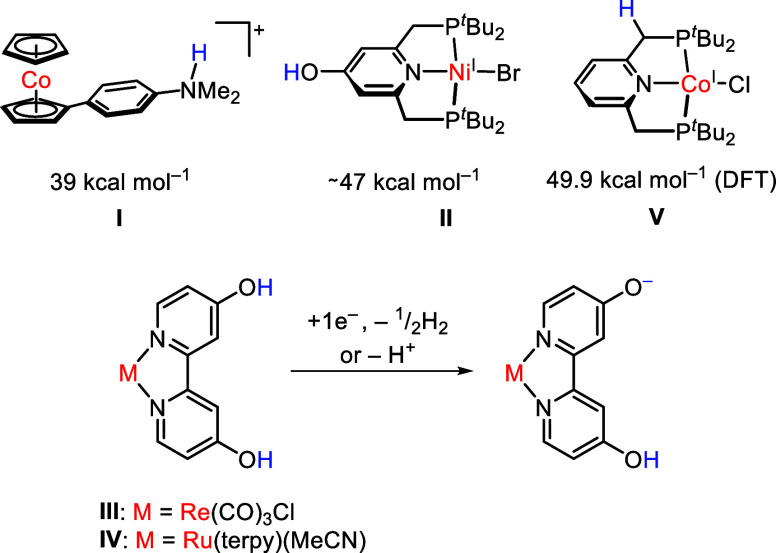
Top: representative 1H^+^/1e^–^-transfer reagents with low BDFE, in which the redox and proton sites are spatially separated; bottom: O–H bond breaking and H_2_ evolution in metal complexes bearing 4-oxo-4*H*-pyridin-1-ide/pyridinol ligands.

We recently introduced a PNP pincer-type ligand with a protic OH function in the ligand backbone and showed that the resulting Ni­(I) complex **II** has a rather low O–H BDFE of 47 kcal mol^–1^ (Figure 1).^7^ Therein, we combined the rigid, strong field pincer-type ligand motif with 3d-metal ions because nickel, cobalt, iron, and manganese complexes with such functional pincer ligands have been successfully utilized in several organic transformations, e.g., thermal (de)­hydrogenation reactions.^8^ **II** is a potent H atom transfer reagent and reduces, e.g., MeC≡N. H_2_ loss was only observed in rather inert solvents such as THF and in the absence of reactive substrate. **II** was prepared in situ by (electro)­chemical reduction of the nickel­(II) ion, which leads to the O–H bond weakening. O–H bond weakening upon reduction and subsequent H_2_ evolution from the ligand backbone has precedents in complexes with 4-oxo-4*H*-pyridin-1-ide/pyridin-4-ol ligands.^9^ Upon one-electron reduction of **III** or **IV**, H_2_ evolution and formation of the anionic ligand was observed (Figure 1, bottom). DFT calculations have been conducted to rationalize the mechanism: ligand-based one-electron reduction of **III** induces a sequence of ligand protonation and, in turn, ligand dearomatization followed by disproportionation, H_2_ evolution, and proton release. The reaction rate constant for the consumption of **III** after electrochemical reduction was determined to be *k*_1_ = 8 s^–1^. Notably, the initial reductions in **III** and **IV** are ligand- rather than metal-based, whereas in **II**, it is nickel-based, and thus, a similar pathway for H_2_ evolution in **II** seemed unlikely. For a related Fe­(II) PCP pincer-type complex with a 4-oxocyclohexa-2,5-dien-1-ide-type core, the reverse reaction, that is, H_2_ splitting, is thermodynamically favored.^10^

This prompted us to investigate the mechanisms of H-atom transfer and undesired H_2_ evolution of such 1H^+^/1e^–^-transition metal transfer reagents with low BDFE, as this could give valuable insights for the design of hydrogenation electrocatalysts. Since **II** is rather reactive, we focused on the corresponding Co­(I) complex.

## Results and Discussion

### Complex Synthesis and Characterization

The ligand HL^PNP^ was synthesized as reported previously.^7^ Deprotonation of the ligand and subsequent reaction with CoCl_2_ yielded the complex [Co­(L^PNP^)­Cl], **1**, as a blue solid in 65% yield after recrystallization. LIFDI-MS (liquid injection field desorption ionization mass spectrometry) confirmed the formation of the complex, and elemental analysis verified the purity (Scheme 1). The Co­(II) complex **2H** ^**2+**^, [Co­(HL^PNP^)­(MeCN)_2_]^2+^, was isolated as an orange-brown solid with BF_4_^–^ counterions in 86% yield in the reaction of [Co­(MeCN)_6_]­(BF_4_)_2_ and HL^PNP^ at rt in THF (Scheme 1). The ESI-MS of **2H** ^**2+**^ displays signals at *m*/*z* 469.0, *m*/*z* 510.1, and *m*/*z* 255.6 corresponding to [Co­(L^PNP^)]^+^, [Co­(L^PNP^)­(MeCN)]^+^, and [Co­(HL^PNP^)­(MeCN)]^2+^, respectively. IR spectroscopy of **2H** ^**2+**^ shows a broad band at a frequency of 3310 cm^–1^, which could be assigned to the OH vibration. The two bands at 2314 and 2286 cm^–1^ are indicative for nitrile vibrations, pointing toward the presence of two acetonitrile ligands in the complex. The elemental analysis was consistent with the formulation as [Co­(HL^PNP^)­(MeCN)_2_]­(BF_4_)_2_. The 5-fold coordination of the Co­(II) ion in MeCN solution was further supported by EPR data, which were collected in frozen MeCN solution at 147 K. Simulation leads to *g*_x_ = 2.00, *g*_y_= 2.28, and *g*_z_ = 2.33 with hyperfine coupling constants of *A*_x_ (1x^59^Co) = 263, *A*_y_ (1x^59^Co) = 20, and *A*_z_(1x^59^Co) = 85 MHz (Figure S27). The *g*_x_ and *A*_x_ hyperfine coupling constants are similar to those determined for the parent complex [Co­(L^2^)­(MeCN)_2_]^2+^ (*cf. g*_1_ = 2.28, *g*_2_ = 2.02, *A* = 94 G, L^2^ = 2,6-bis­((di-*tert*-butylphosphaneyl)-methyl)­pyridine),^11^ whereas planar Co­(II) complexes with similar pincer ligands often exhibit a large *g* anisotropy.^6a,12^ The UV/vis spectrum of **2H** ^**2+**^ shows absorption bands at 323 and 438 nm, which are attributed to π–π* and d–d transitions, respectively. Attempts to crystallize **2H** ^**2+**^ were not successful, and thus, **2H** ^**2+**^ was reacted with NaBPh_4_ to exchange the counterions. Single crystals suitable for an X-ray diffraction study were obtained by diffusion of Et_2_O into this THF solution. However, the refinement revealed that substitution at the ligand backbone occurred during crystallization forming, **3** ^**BPh4**^ [Co­(BPh_3_L^PNP^)­(MeCN)_2_]­(BPh_4_). NaBPh_4_ was likely contaminated with NaB­(OH)­Ph_3_, which reacted with **2H** ^**2+**^ in a condensation reaction.

**Scheme 1:**
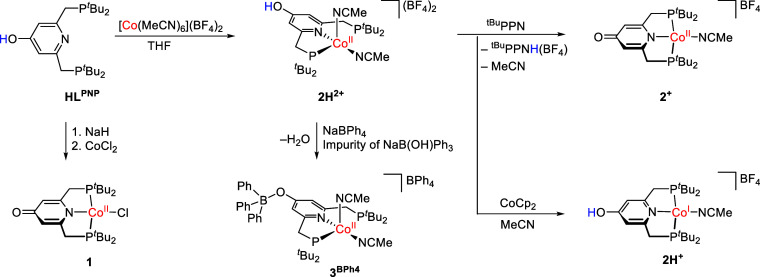
Synthesis of the Complexes Studied in This Work (^tBu^PPN = *N-tert*-Butyl-1,1,1-tri­(pyrrolidin-1-yl)-λ^5^- phosphanimine).

The results of the refinement can be found in Figure 2. Selected bond lengths and angles of **3** ^**BPh4**^ are summarized in Table 1, and further details can be found in the Supporting Information (SI). The cobalt ion is coordinated by two MeCN ligands as well as the PNP ligand. The *τ* value of the cobalt ion equals 0.35, which points to a distorted square-pyramidal coordination geometry.^13^ This can be attributed to the steric bulk of the *tert*-butyl groups. The geometry and the Co–N and Co–P distances are similar to previously reported metal complexes with neutral PNP-pincer-type ligands.^7,11,14^

**Figure 2:**
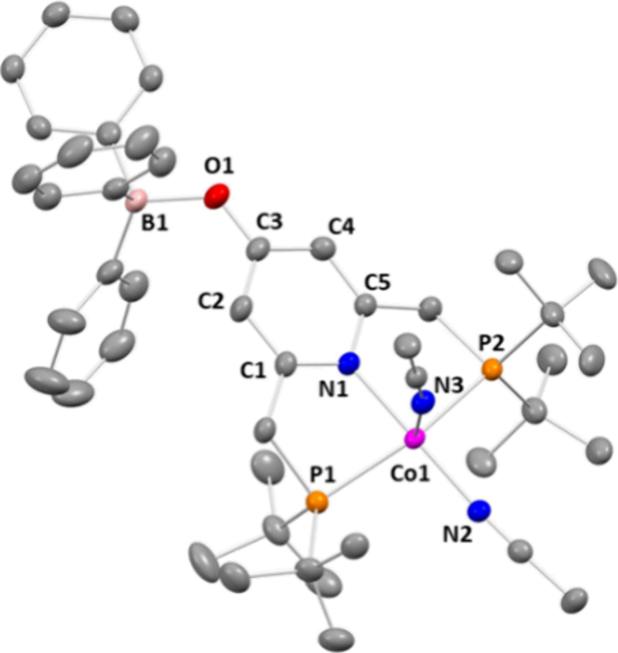
Molecular structure of **3** ^**BPh4**^. Hydrogen atoms, solvent molecules, and the counterion were omitted for clarity. Thermal ellipsoids were set at the 50% probability level.

**Table 1:** Selected Bond Lengths (Å) and Angles (°) for 3^**BPh4**^ with Estimated Standard Deviations in Parentheses

distance	3^BPh4^	angle	3^BPh4^
Co–N1	1.955(2)	N2–Co1–N1	179.30(11)
Co–P1	2.282(9)	N2–Co1–N3	93.12(10)
Co–P2	2.288(9)	N1–Co1–N3	87.51(10)
Co–N2	1.898(3)	P1–Co1–P2	158.48(3)
Co–N3	2.094(3)		

The corresponding Co­(II) complex, **2** ^**+**^ with the deprotonated ligand, was synthesized by reacting **2H** ^**2+**^ with *N*-*tert*-butyl-1,1,1-tri­(pyrrolidin-1-yl)-λ^5^-phosphanimine (^tBu^PPN, Scheme 1). ESI-MS displays a signal at *m*/*z* 510.2, which is assigned to [Co­(L^PNP^)]^+^, **2** ^**+**^. In contrast to **2H** ^**2+**^, which exhibits two stretching vibrations for the acetonitrile ligands, the IR spectrum of **2** ^**+**^ shows only one stretching vibration at 2200 cm^–1^, pointing to the loss of one MeCN ligand upon deprotonation. The elemental analysis was also consistent with the formulation as [Co­(L^PNP^)­(MeCN)]­(BF_4_) with only one MeCN ligand. Magnetic susceptibility measurement in MeCN-*d*_3_ using Evan’s method reveals a spin state of *S* = ^1^/_2_ for **2** ^**+**^, as expected for square planar Co­(II) complexes with a strong field PNP pincer-type ligand (*μ*_eff_ = 2.2 μB at room temperature).^6a,15^ The UV/vis spectrum of **2** ^**+**^ shows bands at 318 and 424 nm. Both bands are blue-shifted compared to **2H** ^**2+**^ due to the dearomatization of the pyridinol forming 4-oxo-4*H*-pyridin-1-ide. Since the structurally similar Co­(I) complex **V** (Figure 1) can be deprotonated with rather strong bases such as KO^*t*^Bu at the CH_2_ unit, we also considered this as a plausible deprotonation site in **2** ^**+**^ (tautomer **2** ^**′+**^, Scheme 2). However, the absence of an OH band at 3310 cm^–1^ in the IR spectrum of **2** ^**+**^ points to a deprotonation at the ligand backbone rather than at the CH_2_ unit (Figure S28). In order to support this structural assignment and the spectroscopic features, DFT calculations were carried out (see Experimental and Computational Details). Simulated UV/vis excitations obtained by TD-DFT calculations were carried out using ORCA. The calculated UV/vis spectrum matches favorably with the experimental spectrum of **2** ^+^ supporting deprotonation at the OH site (Figure S34). Furthermore, in line with the experimental observations, deprotonation at the pyridinol moiety in **2H** ^**2+**^ under simultaneous loss of one acetonitrile ligand is thermodynamically favored over deprotonation at the methylene unit (tautomer **2** ^**′+**^, Scheme 2). Calculations support the experimentally determined low-spin configuration of **2** ^**+**^, with *S* = ^1^/_2_ being ca. 20 kcal mol^–1^ lower in energy than *S* = ^3^/_2_.

**Scheme 2:**
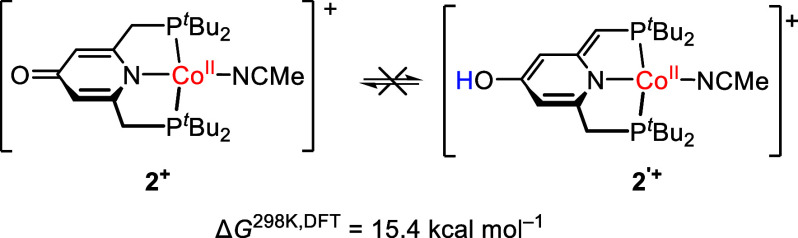
Hypothetical Tautomer 2^**′+**^ of 2^**+**^, which Was Excluded Experimentally and by DFT Studies

### Thermochemical PCET Data

The CV data of **1** in DMF show a reduction peak at *E*_p,c_ −1.73 V with an associated, broad reoxidation process at *E*_p,a_ −1.52 V (*ν* = 0.1 V s^–1^, Figure S24). All CV data are measured with a glassy carbon electrode and referenced internally vs. the FeCp_2_^+|0^ couple (Cp^–^ = cyclopentadienido). The reduction shifts with increasing scan rates pointing to a coupled chemical reactivity, likely chloride loss. Indeed, in the presence of 0.1 M ^*n*^Bu_4_NCl as an electrolyte, the process gets slightly more reversible (Figure S24). The redox potential of the Co^II|I^ couple is about −0.2 V shifted compared to **V** (*cf*. Figure 1), which exhibits a reversible Co^II|I^ oxidation at −1.32 V in THF. The CV data of **2** ^**+**^ in MeCN shows a quasi-reversible reduction at *E*_p,c_ −1.25 V with an associated reoxidation process at *E*_p,a_ −1.12 V, as well as further irreversible oxidation processes at 0.80 and 0.99 V (*ν* = 0.1 V s^–1^, Figure 3 and Figure S 26).

**Figure 3:**
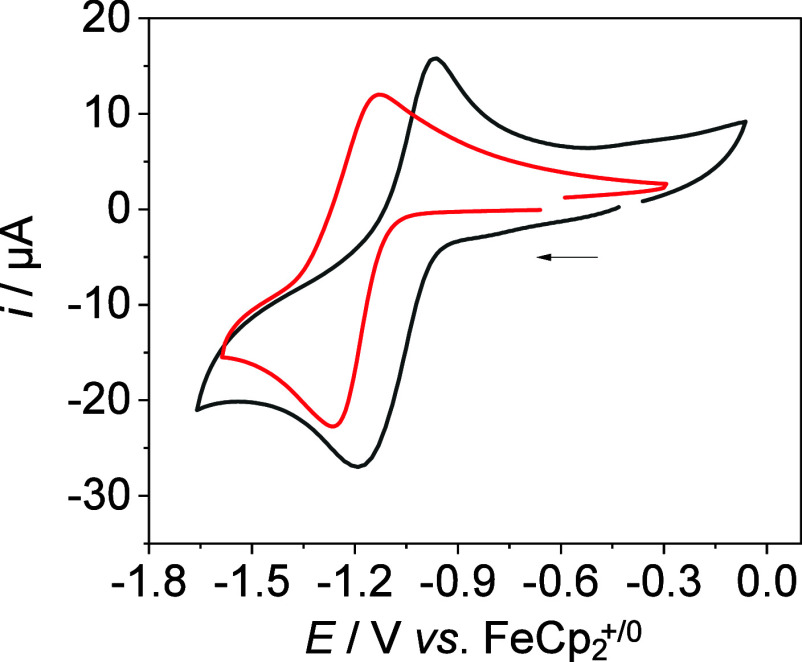
CV data of **2^+^** (red, *c* ∼ 1 mM) and **2H^2+^** (black, *c* ∼ 1 mM) in MeCN, *I* = 0.1 M ^n^Bu_4_NPF_6_, *ν* = 0.1 Vs^–1^.

The potential of **2** ^**+**^ is largely shifted toward less negative potential with regard to **1** likely due to the cationic nature of the complex. The reduction event is in a typical range for cobalt-centered processes.^16^ UV/vis-spectroelectrochemical (UV/vis-SEC) investigation supports (electro)­chemical reversibility of the Co^II|I^ reduction. Reduction of **2** ^**+**^ in MeCN leads to a new prominent band at 560 nm and a shoulder at 375 nm (Figure S6). Furthermore, the data show an isosbestic point at 351 nm. Redox titration with CoCp_2_ confirmed a one-electron nature of the initial reduction (Figure S6). The CV data of **2H** ^**2+**^ show a quasi-reversible reduction at *E*_p,c_ = −1.12 V with a reoxidation at *E*_p,a_ = −1.04 V, and an irreversible oxidation at 1.30 V (*ν* = 0.1 V s^–1^, Figure S25). The reduction is likely coupled to MeCN loss as Co­(I) complexes with such PNP ligands usually exhibit a square planar coordination geometry.^11,17^ Electrochemical reduction of **2H** ^**2+**^ was further investigated by UV/vis-SEC. Upon reduction of **2H** ^**2+**^, a new band appears at 581 nm with a shoulder at 490 nm (Figure S5). The isosbestic point at 354 nm points to a concerted one-step process of reduction and solvent loss forming **2H** ^**+**^. The reduction is reversible and reoxidation of **2H** ^**+**^ leads to the original spectrum of **2H** ^**2+**^ in almost quantitative yield (Figure S5). In order to rationalize the UV/vis feature of **2H** ^**+**^, we also carried out TD-DFT calculations, which are in reasonable agreement with the experimental spectrum (Figure S33). DFT calculations suggests a low-spin configuration of **2H** ^**+**^.

As for previously reported nickel and molybdenum complexes with this PNP ligand, we observe only a small potential difference between **2H** ^**2+**^ and **2** ^**+**^, which indicates a weak thermodynamic coupling between the cobalt ion and the protonation site (Figure 3).^7,18^

Subsequently, we estimated the p*K*_a_ value of the OH proton in **2H** ^**2**+^. Therefore, we added various buffer systems with pH values between 14.5 and 30.0 to a solution of **2H** ^**2**+^. No deprotonation, that is, change in the absorption of **2H** ^**2**+^, was observed for acids with a p*K*_a_ below 16.9, while deprotonation was observed with buffer systems having a pH above 18.8 (Figure S4). This means **2H** ^**2+**^ has a p*K*_a_ of 18 ± 1. Surprisingly, the p*K*_a_ is slightly higher than in the corresponding neutral Ni complex **II**, indicating that neither the overall complex charge nor the metal ion electron configuration has a large impact on the p*K*_a_ but mainly its Lewis acidity. Addition of 5 equiv of buffer solutions with very strong bases lead to additional changes in the UV/vis data of **2H** ^**2**+^ (besides the formation of **2** ^**+**^), presumably due to the second deprotonation at the methylene unit, as observed in **V** (Figure 1).

Having determined the thermochemical data of **2H** ^2+^, we calculated the BDFE of the OH bond according to Bordwell’s equation, where *c*_G_ represents the H^+^/H^•^ reduction potential vs. FeCp_2_^+|0^ in MeCN (52.6 kcal mol^–1^).^20^

$$BDFE=FE^{0}-RT\ln K_{a}+c_{G}$$ 1

$${BDFE}^{RT}=23.06E^{0}+1.364pK_{a}+c_{G}$$ 2

This leads to a BDFE of 52 kcal mol^–1^ for the OH bond in **2H** ^**+**^ at rt. Furthermore, we calculated the p*K*_a_ of **2H** ^**+**^ according to Hess’ law, which equals ∼20 (Scheme 3).

**Scheme 3:**
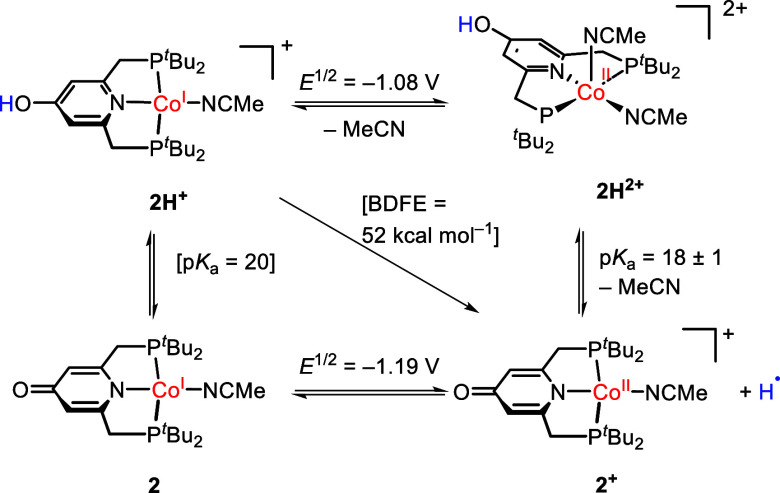
Thermodynamic Square Scheme of the Cobalt Complex^a^

a The values in brackets are calculated values according to Hess’s Law.

We also assessed the BDFE computationally using the isodesmic reaction between **2H** ^**+**^ and TEMPO^•^. Taking the value of 66 kcal mol^–1^ for the BDFE of TEMPOH,^20^ the reaction energy was computed using several density functionals provided in Table S8. The calculated values of the BDFE are in the range of 50.9 to 55.8 kcal mol^–1^, which substantiates the experimentally determined value. The OH BDFE of the nickel complex **II** is about 5 kcal mol^–1^ lower than that of **2H** ^**+**^, because the nickel­(II) complex exhibits a slightly lower reduction potential and is slightly more acidic.

### PCET Reactivity of **2H^+^**

The BDFE of **2H** ^**+**^ equals the BDFE of ^1^/_2_ H_2_ (*cf*. = 52.0 kcal mol^–1^),^5^ and thus, the complex should be prone to H_2_ loss forming **2** ^**+**^. Indeed, **2H** ^**+**^ is not stable over time. The bands at 581 and 490 nm vanish within several hours at rt, and the resulting UV/vis spectrum resembles the one of **2** ^**+**^ (Figure 4 and Figure S 7). Analysis of the gas phase of the headspace of the reaction flask via gas chromatography after chemical reduction of **2H** ^**2+**^ using CoCp_2_ confirms the formation of H_2_ (Figure S29).

**Figure 4:**
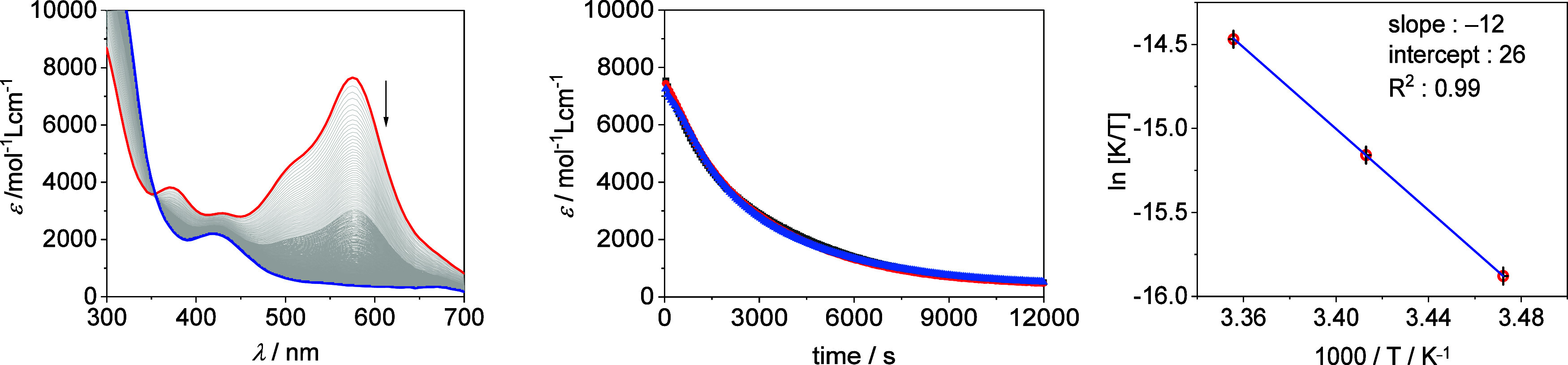
Left UV/vis spectra of **2H^+^** (red) over time (end: blue), *c*_0_ = 0.06 mM. Middle: change of the extinction at 581 nm of **2H** ^**+**^ over time, *c*_0_ = 0.06 mM (black), 0.075 mM (red), and 0.125 mM (blue). Right: Eyring plot of the H_2_ evolution From **2H^+^**.

Since we also aimed to elucidate mechanistic details of the H_2_ evolution reaction, we conducted kinetic investigations. At first, we studied the decay of the characteristic absorption band of **2H** ^**+**^ at 581 nm by UV/vis spectroscopy in dependence of the concentration of **2H** ^**+**^ (Figures S8 and S9). All plots show isosbestic points at 354 nm. Thus, we assume a clean conversion of **2H** ^**+**^ forming **2** ^**+**^. Surprisingly, the half-life of **2H** ^**+**^ was independent of its concentration, which means that the reaction rate is first-order with respect to **2H** ^**+**^ (Figure 4 and Figure S10). This argues against the most intuitive reaction mechanism for H_2_ formation, that is, a bimolecular reaction of 2 equiv **2H** ^**+**^ forming H_2_ and 2 equiv of **2** ^**+**^. Approximation of the initial rate of the reaction from the various concentration-dependent measurements confirmed this (Figure S11). The rate constant *k* was determined from the slope of the linearization plots over two half-lives, and it equals 3.2 × 10^–4^ s^–1^ at 25 °C (Table 2, Figure S11, and Table S5). Notably, the reaction rate for consumption of **2H** ^**+**^ is four orders of magnitude slower than in the Re complex **III.**

**Table 2:** Rate Constants for the Decay of **2H^+^** in Dependence on the Concentration and the Temperature

*c***_2H_^+^** (mM)	*k* (s^–1^)^a^	*T* (K)	standard deviation
0.06	3.2 × 10^–4^	298	0.3 × 10^–4^
0.075	3.1 × 10^–4^	298	0.3 × 10^–4^
0.125	3.3 × 10^–4^	298	0.1 × 10^–4^
0.075	1.5 × 10^–4^	293	0.2 × 10^–4^
0.075	0.73 × 10^–4^	288	0.09 × 10^–4^

a Average value of three independent runs.

Subsequently, temperature-dependent measurements were conducted to derive the activation parameters. As expected, the reaction rate decreases with decreasing temperature (Table 2, Figures S14 and S15, and Table S6). Eyring analysis revealed an activation enthalpy of Δ*H* ^‡^ 24.3 ± 0.2 kcal mol^–1^ and activation entropy Δ*S* ^‡^ of 9.4 ± 0.6 cal mol^–1^ K^–1^ (Figure 4), and in turn, an activation barrier Δ*G* ^‡^ of 21.2 ± 0.3 kcal mol^–1^ at 25 °C. The reaction is under enthalpic control at rt as Δ*H* ^‡^ > *T*Δ*S* ^‡^. The positive activation entropy also points against a bimolecular reaction of 2 equiv **2H** ^**+**^ forming H_2_ and 2 equiv of **2^+^** as rate determining step.

To account for the first-order kinetics in **2H** ^**+**^ and the positive entropy, we hypothesize as the first step an *irreversible*, intramolecular proton transfer from the OH unit to the metal center forming a Co­(III) hydride species, **2′H** ^**+**^ (Scheme 4). The proposed hydride then rapidly reacts with **2H** ^**+**^ forming 2 equiv of **2** ^**+**^ and 1 equiv of H_2_, and thus, its steady-state concentration is negligible.

**Scheme 4:**
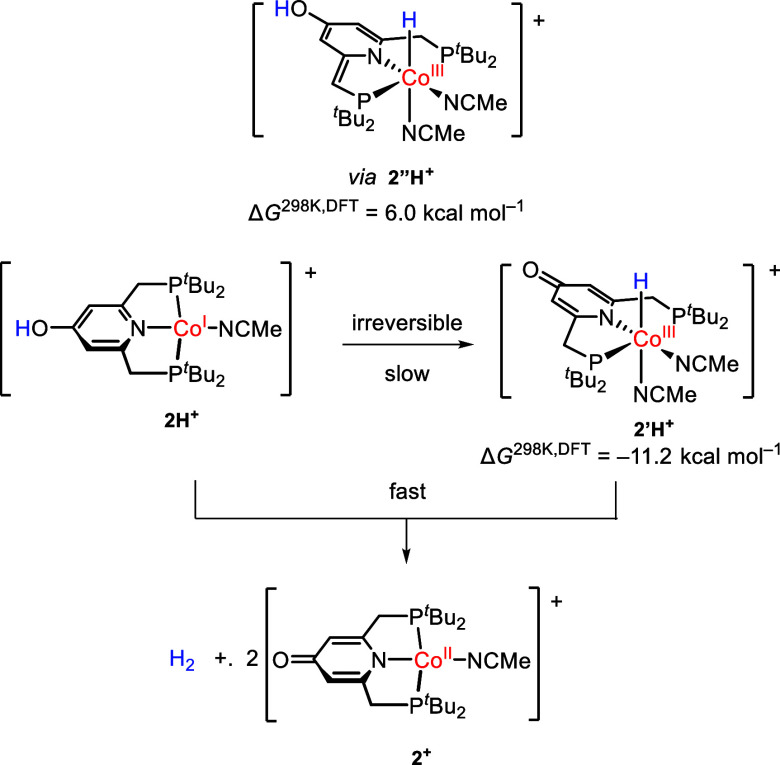
Proposed Mechanism of H_2_ Evolution Based on DFT Calculations and Kinetic Analysis

Indeed, DFT calculations predicted that the conversion of **2H** ^**+**^ to the putative Co­(III) hydride isomer **2′H** ^+^ is thermodynamically favored by −11.2 kcal mol^–1^. To account for the additional acetonitrile coordination in addition to the hydride shift, **2H** ^**+**^ was optimized in the presence of an additional explicit solvent molecule (Figure S35). Comparing the two potential coordination positions of the H atom, the *cis* hydride conformation is favored over *trans* by 21.7 kcal mol^–1^.

The estimated distance between the OH proton and a hydride in an apical position is around 7 Å, which is rather large. Thus, we surmised that the proton shift could appear via the methylene unit forming the isomer **2″H** ^**+**^ (Scheme 4). Indeed, DFT calculations revealed that **2″H** ^**+**^ is 6.0 kcal mol^–1^ higher in energy than **2H** ^+^, supporting that it could be a plausible intermediate. In order to probe for this scenario, labeling experiments were conducted using **2D** ^+^. ^2^H NMR analysis following the reduction of **2D** ^**2+**^ with 1 equiv of CoCp_2_ revealed distinct peaks at 5.0 ppm (dihydrogen) and 3.5 ppm (Figure S32). The latter peak has been assigned to a deuterium atom at the methylene unit of the pincer ligand, which experimentally supports intramolecular proton transfer via the methylene unit.^6a^

Since the hydrogen evolution from **2H** ^**+**^ is rather slow, we were intrigued to know if it is possible to outcompete H_2_ evolution by direct H atom transfer to suitable acceptors. Indeed, the reaction of 2,4,6-*tri-tert*-butyl phenoxy radical (BDFE of 74.8 kcal mol^–1^)^20^ with **2H** ^**+**^ is facile and we observe a rather clean formation of the corresponding phenol. NMR spectroscopy reveals a 90% spectroscopic yield of 2,4,6-*tri-tert*-butyl phenol (Figure S30), and the isosbestic point at 354 nm in the UV/vis spectra upon gradual addition of the radical indicates no secondary reaction (Figure S16). Stopped-flow measurements at −35 °C showed that the reaction of equimolar amounts of 2,4,6-*tri-tert*-butyl phenoxy radical with **2H** ^**+**^ was accomplished within less than 1 ms, which excludes formation of the putative hydride species as intermediate but rather a direct transfer of the H atom. However, the fast reaction excluded further kinetic investigation. Subsequently, we investigated the H atom transfer to TEMPO^•^ (BDFE of 66 kcal mol^–1^).^20^ The isosbestic point at 354 nm in the UV/vis spectra upon adding TEMPO^•^ to a solution of **2H** ^**+**^ confirmed clean conversion (Figure S17). ^1^H NMR spectroscopy revealed a spectroscopic yield of 92% for TEMPOH (Figure S17). These reactions exemplify that H atom transfer reactions to organic O-radical compounds are very fast and selective for **2H** ^**+**^ ^7^ leading to the products in high spectroscopic yields.

The reaction of **2H** ^**+**^ with TEMPO^•^ was slightly slower than with the phenoxy radical (several ms), and thus, we conducted double-mixing stopped-flow measurements to gain further insight into the reaction. **2H** ^**2+**^ and CoCp_2_ were mixed to form **2H** ^**+**^ *in situ* and subsequently reacted with TEMPO^•^. The decrease of the characteristic band of **2H** ^**+**^ at 585 nm was monitored under pseudo first-order conditions with 10, 15, 20, and 30 equiv of TEMPO^•^ at −35 °C (Figure S18). Reactions at higher temperatures or higher TEMPO^•^ concentrations were too fast to be monitored reliably. The pseudo-first-order reaction rate constants *k*_obs_ have been determined from the pseudo-first-order linearization plots of the concentration-dependent measurements at −35 °C (Figures S19–S22). The values are shown in Table 3. The plot of *k*_obs_ versus the concentration of TEMPO^•^ leads to the second-order rate constant *k*_2_ = 1.3 ± 0.3 × 10^4^ s^–1^ M^–1^ at −35 °C for the reaction of **2H** ^**+**^ and TEMPO^•^ in MeCN (Figure S22). The plot exhibits an intercept of about 34, indicating a side reaction first order in **2H** ^**+**^, likely decomposition due to traces of moisture.

**Table 3:** Pseudo-First-Order Rate Constants for the Reaction of **2H^+^** (*c* = 0.45 mM) with TEMPO^•^, MeCN, −35 °C, Average of Three Independent Runs

*c*_TEMPO_^•^ (mM)	*k*_obs_ (s^–1^)	std. dev. (s^–1^)
4.5	0.93 × 10^2^	0.03 × 10^2^
6.75	1.1 × 10^2^	0.3 × 10^2^
9.0	1.5 × 10^2^	0.2 × 10^2^
13.5	2.0 × 10^2^	0.1 × 10^2^

From the second-order reaction rate constant *k*_2_ of about 10^4^ s^–1^ M^–1^, we estimated an activation barrier Δ*G* ^‡^ of ∼12 kcal mol^–1^ for the reaction of **2H** ^**+**^ and TEMPO^•^ according to transition state theory (eq 3).

$$k_{2}=\frac{k_{B}T}{h}e^{-\Delta G^{‡}/RT}$$ 3

Since the activation barrier is much lower than the activation barrier determined for the putative hydride formation, this species can be safely excluded as kinetically relevant intermediate. Furthermore, it suggests a concerted PCET reaction between **2H** ^**+**^ and TEMPO^•^ rather than stepwise PT/ET or ET/PT mechanism based on thermodynamic considerations (Scheme 5): In case of a stepwise transfer, the free energy for PT or ET intermediate formation represents a lower limit for the activation barrier of the overall reaction (assuming no additional kinetic barriers for the steps), as their formation represents the initial step in a stepwise PT/ET or ET/PT process, respectively. An initial PT intermediate forming **2** and TEMPOH^+^ would be uphill by about 29 kcal mol^–1^ as reasoned from the p*K*_a_ of TEMPOH^+^ with 41 and of **2H** ^**+**^ with 20.^19^ An initial ET intermediate forming **2H** ^**2+**^ and TEMPO^–^ would be uphill by about 20 kcal mol^–1^, as estimated from *E* ^0^ of TEMPO^•|−^ with −1.95 V^19^ and of **2H** ^**2+|+**^ with −1.08 V. Thus, formation of the intermediates can be safely excluded as their formations are both much higher in energy than the estimated activation energy of the H atom transfer reaction.

**Scheme 5:**
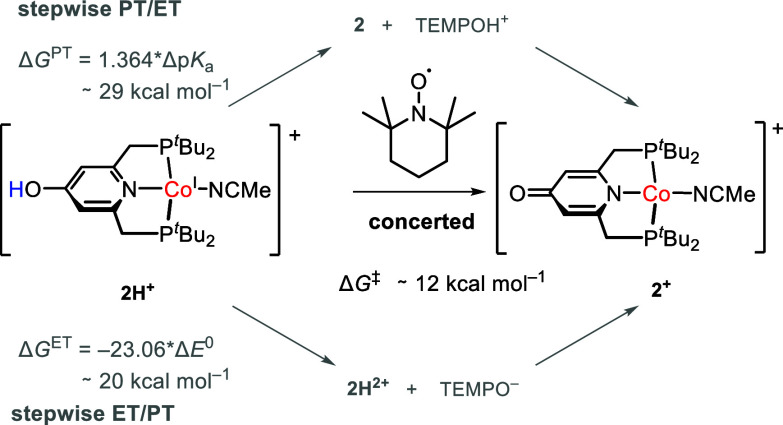
Stepwise vs. Concerted H-Atom Transfer from **2H^+^** to TEMPO^•^ in MeCN

## Conclusions

We investigated the PCET reagent **2H** ^**+**^, which consists of a redox active Co­(II|I) metal ion combined with a proton-responsive PNP pincer ligand. The complex has a weak OH bond with a BDFE of 52 kcal mol^–1^, as assessed experimentally and computationally. In line with this, it slowly loses H_2_ with a reaction rate constant of 3.2 × 10^–4^ at rt. Kinetic analysis revealed a first-order dependence in **2H** ^**+**^ and a positive activation entropy Δ*S* ^‡^ of 9.4 ± 0.6 cal mol^–1^ K^–1^ for H_2_ loss, which argues against a bimolecular pathway. Based on further labeling and computational studies, an irreversible, intramolecular proton shift *via* the methylene unit of the pincer ligand is proposed, forming a Co­(III)-hydride species **2′H** ^**+**^ as an isomer of **2H** ^**+**^, which readily reacts with a second equivalent of **2H** ^+^ forming H_2_ and **2** ^**+**^. Due to the spatial separation of the proton and electron acceptor site, H_2_ formation is very slow and can be easily outcompeted by H atom transfer from **2H** ^**+**^ to organic radical species such as TEMPO^•^ or 2,4,6-*tert*-butylphenoxyl radical. Kinetic analysis revealed that the reaction rate constant is eight orders of magnitude higher (*cf*. *k*_2_ of 10^4^ s^–1^ M^–1^) for the reaction of **2H** ^**+**^ with TEMPO^•^, emphasizing that H_2_ loss from **2H** ^**+**^ can be successfully suppressed in the presence of suitable acceptors. Formation of **2H** ^**+**^ can be accomplished with moderately strong reducing reagents at a potential of only −1.08 V and with moderately strong acids (p*K*_a_ = 18 ± 1), which makes it promising for electrochemically driven PCET reactions.

## Experimental and Computational Details

All manipulations were carried out by means of common Schlenk-type techniques involving the use of a dry argon or nitrogen atmosphere or performed in an MBraun glovebox. Solvents were dried using a MBraun Solvent Purification System and stored over molecular sieves 3 Å. [Co­(MeCN)_6_]­(BF_4_)_2_ ^21^ and HL^PNP^ ^7^ were prepared according to literature-known procedure.

### [Co^II^(L^PNP^)­Cl] (**1**)

2,6-Bis­(*di-tert*-butylphosphinomethyl)-4-hydroxypyridine (165 mg, 0.40 mmol, 1.00 equiv) and NaH (12.0 mg, 0.5 mmol, 1.25 equiv) were stirred in thf (4 mL) for 2 h until a clear solution was obtained. CoCl_2_ (51.9 mg, 0.40 mmol, 1.00 equiv) was then added, and the mixture was stirred overnight. The solvent was removed, and the complex was extracted with DCM. After washing with hexane, the product was obtained as a purple, crystalline solid (137 mg, 0.26 mmol, 66%). LIFDI-MS [C_23_H_42_ClCoNOP_2_]^+^: *m*/*z =* 505.7. Elemental analysis calc. for C, 54.6; H, 8.57; N, 2.77. Found: C 54.9; H 8.55; N 2.71.

### [Co^II^(HL^PNP^)­(MeCN)_2_]­(BF_4_)_2_ (**2H^2+^**)

HL^PNP^ (100 mg, 0.24 mmol, 1 equiv) and [Co­(MeCN)_6_]­(BF_4_)_2_ (116 mg, 1 equiv) were stirred for 12 h in thf (5 mL) at rt. Subsequently, the solvent was removed *in* *vacuo*. Recrystallization from DCM/hexane and MeCN/Et_2_O afforded **2H** ^**2+**^ with two BF_4_^–^ counterions as an orange powder. Yield: 150 mg (86%). Elemental analysis calc. for C_27_H_49_CoN_3_OP_2_B_2_F_8_: C, 44.6; H, 6.8; N, 5.79. Found: C, 44.9; H, 6.6; N, 5.8. *ESI*-MS [C_27_H_49_CoN_3_OP_2_]^+^ ([M – H]^+^): *m*/*z* = 551.1, [C_25_H_46_CoN_3_OP_2_]^+^ ([M-MeCN-H]^+^): *m*/*z* = 510.2. IR­(KBr) (ν/cm^–1^): 3309 (br), 2966 (m), 2314 (w), 2286 (w), 1624 (s), 1475 (s), 1365 (m).

X-ray quality crystals of **3** ^**BPh4**^ were grown by slow diffusion of Et_2_O to an acetonitrile solution of **2H** ^**2+**^ and 2 equiv of NaBPh_4_.

### [Co^II^(L^PNP^)­(MeCN)]­BF_4_ (**2^+^**)

**2H** ^**(BF4)2**^ (100 mg, 0.136 mmol, 1 equiv) was dissolved in 5 mL of MeCN, and *tert*-butylimino-tri­(pyrrolidino)­phosphoran (43 μL, 0.136 mmol, 1 equiv) was added; the solution was stirred for 12 h at rt. Subsequently, the solvent was removed *in* *vacuo*. Recrystallization from MeCN/Et_2_O afforded **2** ^**+**^ with a BF_4_^–^ counterion as brown powder. Yield: 61 mg (74%). Elemental analysis calc. for C_25_H_45_CoN_2_OP_2_BF_4_: C, 50.2; H, 7.6; N, 4.7%. Found: C, 49.6; H, 7.5; N, 5.2%. IR­(KBr) (ν/cm^–1^): 2966 (w), 2201 (w), 1593 (m), 1475 (m), 1347 (m).

Computations in the present study were performed with the ORCA 5 program package^22^ utilizing our custom-built in-house HPC cluster. Electronic ground-state calculations, including geometry optimizations and frequencies, were carried out with density functional theory (DFT) and the TPSSh meta-hybrid functional^23^ together with Grimme’s D3 dispersion^24^ correction and Ahlrichs’ def2-TZVP basis set.^25^ Implicit solvation was included in all calculations via the CPCM model and acetonitrile as solvent. The resolution of identity approximation (RIJCOSX) was used, along with the corresponding auxiliary basis sets, to accelerate the calculations. Gibbs free energies at 298 K were calculated using the rigid-rotor harmonic oscillator approximation as implemented in ORCA. Time-dependent DFT calculations (TD-DFT) were performed on already obtained geometries using the (spin-component scaled) double-hybrid functional SCS-wPBEPP86 ^26^ and the standard def2-TZVP basis set with *n* = 30 roots, each. Structures were visualized with Chemcraft.^27^

## Supplementary Material





## Notes

Additional experimental details, analytical data, and details on the calculations (PDF)
xyz coordinates of the calculated structures (XYZ)
