# Supplementary material for: Mechanistic Studies of the Proton-Coupled Electron Transfer Reactivity of a Cobalt Complex with a Proton-Responsive PNP Pincer-Type Ligand
Source: Inorg Chem. 2025 Jul 10;64(28):14466–74. doi: 10.1021/acs.inorgchem.5c01792 (PMC12284867; doi:10.1021/acs.inorgchem.5c01792)
Supplement: Supplementary file 1 [file ic5c01792_si_001.pdf]

Supporting Information

Mechanistic Studies of the Proton-Coupled Electron Transfer Reactivity of a Cobalt Complex with a Proton-Responsive PNP Pincer-Type Ligand

Jyotima Mukherjee,[1] Nils Ostermann,[1] Jan Pecak,[2] Matthias Otte,[1] Maren Podewitz[2]* and Inke Siewert[*1,3]

1: Georg-August-Universität Göttingen, Institut für Anorganische Chemie, Tammannstr. 4, 37077 Göttingen, Germany

2: TU Wien, Institute of Materials Chemistry, Getreidemarkt 9, 1060 Wien, Austria

3: Georg-August-Universität Göttingen, International Center for Advanced Studies of Energy Conversion, Tammannstr. 4, 37077 Göttingen, Germany

E-Mail: inke.siewert@chemie.uni-goettingen.de; maren.podewitz@tuwien.ac.at

Table of content

Experimental Section 2
- General 2
- Instrumentation 2
- UV/Vis Spectroscopy Experiments 3
- UV/Vis Spectroelectrochemistry 3
- Stopped Flow Experiments 3
- NMR Spectroscopy Experiments 3
- X-ray Single-Crystal Structure Analysis 4

Crystallographic Details 4

Analytical Data 12
- UV/Vis and UV/Vis SEC Data 12
- Electrochemical Data 22
- EPR Data 23
- IR Data 23
- GC-WLD Data 24
- NMR Data 24

Computational Section 25
- TD-DFT Data 26
- Results of the BDFE Calculations 26
- Potential transformation of **2H**$^+$ to **2'H**$^+$ 27

References 27

Experimental Section

General

All manipulations were carried out by means of common Schlenk-type techniques involving the use of a dry argon or nitrogen atmosphere or performed in an MBraun glovebox. Solvents were dried using a MBRAUN Solvent Purification System and stored over molecular sieves 3 Å. $[Co(MeCN)_6](BF_4)_2$[1] and HL^{PNP}[2] were prepared according to literature known procedures. Benzylammonium triflate, $TMGHPF_6$, $DBUHPF_6$, $Et_3NHBArF$, and $TBDHPF_6$ were prepared following a literature known procedure (TMG = 1,1,3,3-Tetramethylguanidin, DBU = Diazabicycloundecen, $BArF_{24}$ = Tetrakis(3,5-bis(trifluoromethyl)phenyl)borate, TBD = Triazabicyclodecene).[3]

Instrumentation

^{1}H-, ^{13}C- spectra were recorded with a Bruker Avance 300 NMR spectrometer (^{1}H 300 MHz, ^{13}C 75 MHz, ^{31}P 120 MHz) CD_3CN as the solvent at 25°C. The ^{1}H-, ^{13}C-NMR spectra were calibrated against the residual protons and natural-abundance ^{13}C resonances of the deuterated solvent (CD_3CN: δ_H = 1.94 ppm, δ_C = 118.26 ppm). ^{31}P-NMR spectra are reported relative to phosphoric acid as an external reference (δ_P = 0.0 ppm). Signal multiplicities are abbreviated as: s (singlet), d (doublet), t (triplet), q (quartet), m (multiplet), br (broad).

Microanalyses were performed with an Elementar Vario El II elemental analyser.

Mass spectra were recorded using a Bruker APEX IV micrOTOF or a Bruker Autoflex Speed mass spectrometer.

The IR spectra was recorded with a Bruker Invenio-R spectrometer.

The UV/Vis SEC (spectroelectrochemical) data were recorded with a BWTek Exemplar LS spectrometer with a BWTek Deuterium/Tungsten light source, which is connected via fiber patch cables to the glovebox.

The UV/Vis data was recorded with a BWTek Exemplar LS spectrometer or Specord 50 Plus spectrometer from ANALYTIK JENA or in an Agilent Cary 60 spectrometer equipped with an Unisoku Cryostat (CoolSpek).

Headspace analysis to confirm H_2 evolution was conducted with a Shimadzu GC-2014 gas chromatograph with a thermal conductivity detector (TCD). A molecular sieves column (5 Å 80/100) with Ar as carrier gas was used.

For Stopped-flow measurements, the TgK Scientific Hi-Tech KinetAsystTM CSF-61DX2 double-mixing cryo-stopped-flow system was used to collect the kinetic data in single-mixing mode. Using a xenon lamp as the light source and a KinetaScan Rapid Scanning CCD (Charge Coupled Device) detector, UV/Vis spectra in the range of 300-800 nm were collected. All mixing chambers were cleaned with dry, deoxygenated solvent prior to the experiment to guarantee anaerobic conditions.

Electrochemical measurements were recorded with a Gamry Instruments Reference 600+ in dry MeCN under N_2 atmosphere in the glovebox. A common three electrode setup was used with a glassy carbon working electrode (GC: CH Instruments, ALS Japan; A= 7.1 mm^2), a platinum wire as a counter electrode, and a silver wire in silver nitrate solution (I = 0.01 M) in a fritted sample holder as pseudo reference electrode. Spectroelectrochemical grade $^n Bu_4NPF_6$ was dried at 100°C in *vacuo* for 8 h and used as conducting salt, I = 0.1 M. All data were referenced internally *vs*. the $Fc^{+/0}$ redox couple. i*R* compensation was performed by the positive feedback method, which is implemented in the PHE200 software of Gamry.

UV/Vis Spectroscopy Experiments

All UV/Vis experiments were carried out in the N_2-filled glovebox.

Titration of $\mathbf{2H^{2+}}$ with buffer solutions

2 mL of a solution of $\mathbf{2H^{2+}}$ (0.25 mM) in MeCN were placed in a 10 mm path-length quartz cuvette equipped with a stirrer bar and capped with a septum. A solution of the different X/XH buffer system in MeCN (0.05 M) was prepared and added in 10 µL portions via a syringe (10 µL equal 1 equiv), and the spectral changes were recorded (Figure S 4, X = benzosulphonamide, 2,4,6- trimethylpyridine, benzylamine, triethylamine,1,1,3,3-tetramethylguanidin, diazabicycloundecene, triazabicyclodecene).

Kinetics of H_2 Evolution from $\mathbf{2H^{+}}$

The kinetic analysis was done with UV/Vis spectroscopy by recording the change in absorbance of the band at 587 nm every 5 min. The reaction kinetics was measured in different concentrations (0.06, 0.075 and 0.125 mM) at 25 °C and at different temperatures (25°C, 20°C and 15°C, Figure S 7-Figure S 15).

Titration of $\mathbf{2H^{+}}$ with H atom acceptors

2 mL of a solution of $\mathbf{2H^{+}}$ (0.075mM) was prepared in MeCN and placed in a cuvette. A solution of the H atom acceptor (30 µL, 5 mM solution) was added stepwise in 0.2 eq. UV/Vis spectra were recorded in every step at rt under N_2 atm. (Figure S 16-Figure S 17). The experiments took 120 seconds.

UV/Vis Spectroelectrochemistry

A solution of $\mathbf{2H^{2+}}$ (0.75 mM) or $\mathbf{2^{+}}$ (0.1 mM) in MeCN (0.1 M NBu_4PF_6) was added into a thin-layer quartz cuvette (0.1 cm diameter). The cuvette was equipped with a Pt-gauze working electrode, a Pt counter electrode, and a silver wire in silver nitrate solution (I =0.01 M) in a fritted sample holder as pseudo reference electrode. A CV was recorded to determine the potential of the reduction. The solution was removed, and the cell was refilled with a solution of $\mathbf{2H^{2+}}$ or $\mathbf{2^{+}}$ in MeCN. The potential of the reduction was applied and every 10 s a UV/Vis spectrum was recorded until the current dropped down to the capacitive current.

Stopped Flow Experiments

All the solutions were prepared in N_2 atmosphere glovebox and transferred into the stopped-flow spectrometer using gastight syringes. Solutions of $\mathbf{2H^{+}}$ (0.45 mM) and different concentrations of TEMPO$^{\bullet}$ (4.5, 6.75, 9, 13.5 mM) in MeCN were mixed at −35 °C (Figure S 18-Figure S 23). The temperature was maintained by using a liquid-nitrogen-cooled ethanol bath equipped with a cryostat.

NMR Spectroscopy Experiments

Reaction of $\mathbf{2H^{+}}$ with H atom acceptors

A solution of 7 mg of $\mathbf{2H^{+}}$ (0.01 mM) was reduced by adding 1.8 mg (0.01 mM) of $CoCp_2$ in MeCN-d_3. The H atom acceptor reagents (0.01 mM) and 2,4,6 *tri*-methoxy benzene as internal standard were added to this solution. Analysis of the ^{1}H NMR spectra revealed the formation of 2,4,6-tri-tert-butyl phenol (90% yield relative to the internal standard) and TEMPOH (92% yield relative to the internal standard). The yields were quantified by integrating the respective NMR signals against the internal standard (Figure S 30, Figure S 31).

Labelling experiment

A solution of $\mathbf{2^{+}}$ (50 mg, 0.084 mM) was reacted with [ColD][BArf$_{24}$] (82.6 mg, 0.084 mM) in CD_3CN to yield $\mathbf{2D^{2+}}$. Subsequently, $\mathbf{2D^{2+}}$(80 mg, 0.053 mM) was dissolved in 0.5 mL of CH_3CN, and 1 equivalent of cobaltocene (10.1 mg) was added to the solution. The reaction mixture was analysed by ^{2}H NMR spectroscopy to confirm the formation of the desired species (Figure S 32).

X-ray Single-Crystal Structure Analysis

CCDC-2429570 (**3(BPh$_4$)**) contains the supplementary crystallographic data for this paper. This data can be obtained free of charge via http://www.ccdc.cam.ac.uk/products/csd/request/ (or from Cambridge Crystallographic Data Centre, 12 Union Road, Cambridge, CB2 1EZ, UK. Fax: +44-1223-336-033; e-mail: deposit@ccdc.cam.ac.uk)

Crystallographic Details

Suitable single crystals for X-ray structure determination were selected from the mother liquor under an inert gas atmosphere and transferred in protective perfluoro polyether oil on a microscope slide. The selected and mounted crystals were transferred to the cold gas stream on the diffractometer. The diffraction data were obtained at 100 K on a Bruker D8 three-circle diffractometer, equipped with a PHOTON III detector and an INCOATEC microfocus source with Quazar mirror optics (Mo-Kα radiation, λ= 0.71073 Å).

The data obtained were integrated with SAINT and a semi-empirical absorption correction from equivalents with SADABS was applied. The structure was solved and refined using the Bruker SHELX 2014 software package.[4] All non-hydrogen atoms were refined with anisotropic displacement parameters. All C-H hydrogen atoms were refined isotropically on calculated positions by using a riding model with their U_{iso} values constrained to 1.5 U_{eq} of their pivot atoms for terminal sp^3 carbon atoms and 1.2 times for all other atoms.

X-ray Single-Crystal Structure Analysis of **3^{BPh4}**

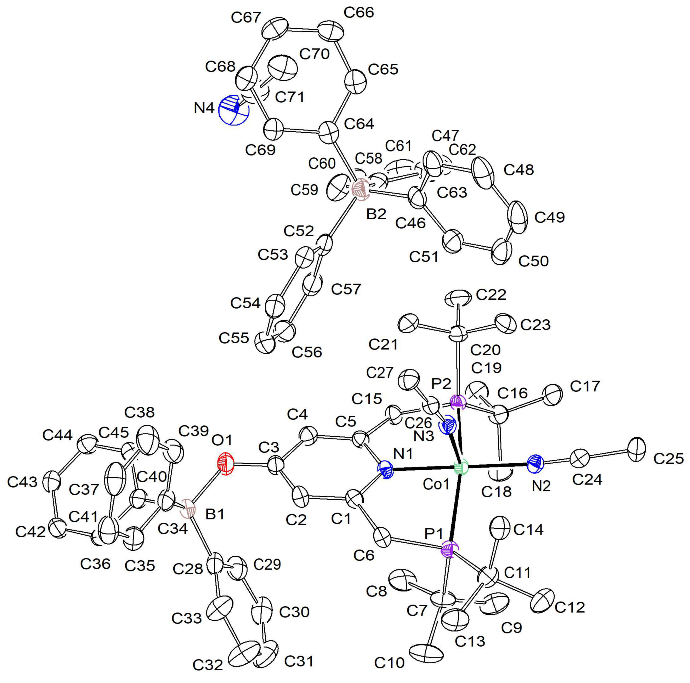

*Figure S 1. Thermal ellipsoid plot of **3^{BPh4}** with the anisotropic displacement parameters drawn at the 50% probability level. The asymmetric unit contains one complex molecule, one counter ion and one solvent molecule.*

Table S 1: Crystal data and refinement details for $\mathbf{3^{BPh4}}$.

Identification code	IS_JM_210122_2_MO	
Empirical formula	$C_{71}H_{86}B_2CoN_4OP_2$	
Formula weight	1153.92	
Temperature	100(2) K	
Wavelength	0.71073 Å	
Crystal system	Monoclinic	
Space group	$P2_1/c$	
Unit cell dimensions	a = 12.8890(7) Å	a= 90°
	b = 23.8838(13) Å	b= 101.382(2)°
	c = 21.2577(12) Å	g = 90°
Volume	6415.2(6) $Å^3$	
Z	4	
Density (calculated)	1.195 Mg/m^3	
Absorption coefficient	0.363 mm^{-1}	
F(000)	2460	
Crystal size	0.154 x 0.094 x 0.080 mm^3	
Crystal shape and color	Block,	clear intense red
Theta range for data collection	1.913 to 25.710°	
Index ranges	-15<=h<=15, -29<=k<=29, -25<=l<=25	
Reflections collected	107817	
Independent reflections	12200 [R(int) = 0.0622]	
Completeness to theta = 25.242°	100.0 %	
Refinement method	Full-matrix least-squares on F^2	
Data / restraints / parameters	12200 / 0 / 745	
Goodness-of-fit on F^2	1.213	
Final R indices [I>2sigma(I)]	R1 = 0.0622,	wR2 = 0.1217
R indices (all data)	R1 = 0.0747,	wR2 = 0.1289
Largest diff. peak and hole	0.481 and -0.326 $eÅ^{-3}$	

Table S 2. Bond lengths [Å] and angles [°] for $\mathbf{3^{BPh4}}$

Co(1)-N(2)	1.898(3)
Co(1)-N(1)	1.955(2)
Co(1)-N(3)	2.094(3)
Co(1)-P(1)	2.2823(9)
Co(1)-P(2)	2.2884(9)
N(1)-C(5)	1.369(4)
N(1)-C(1)	1.370(4)
P(1)-C(6)	1.833(3)
P(1)-C(7)	1.869(3)
P(1)-C(11)	1.878(3)
B(1)-O(1)	1.565(4)
B(1)-C(34)	1.626(5)
B(1)-C(28)	1.629(5)
B(1)-C(40)	1.629(5)
O(1)-C(3)	1.310(4)
C(1)-C(2)	1.375(4)
C(1)-C(6)	1.504(4)
P(2)-C(15)	1.832(3)
P(2)-C(20)	1.865(3)
P(2)-C(16)	1.875(3)
N(2)-C(24)	1.143(4)
B(2)-C(58)	1.641(5)

B(2)-C(46)	1.646(5)
B(2)-C(64)	1.647(5)
B(2)-C(52)	1.647(5)
C(2)-C(3)	1.399(4)
C(3)-C(4)	1.401(4)
N(3)-C(26)	1.139(4)
C(4)-C(5)	1.371(4)
N(4)-C(71)	1.150(5)
C(5)-C(15)	1.501(4)
C(7)-C(8)	1.530(5)
C(7)-C(9)	1.530(5)
C(7)-C(10)	1.538(5)
C(11)-C(12)	1.534(5)
C(11)-C(14)	1.536(4)
C(11)-C(13)	1.542(5)
C(16)-C(17)	1.533(5)
C(16)-C(19)	1.534(5)
C(16)-C(18)	1.537(4)
C(20)-C(23)	1.526(5)
C(20)-C(21)	1.531(5)
C(20)-C(22)	1.534(4)
C(24)-C(25)	1.459(4)
C(26)-C(27)	1.455(4)
C(28)-C(33)	1.389(5)
C(28)-C(29)	1.406(5)
C(29)-C(30)	1.378(5)
C(30)-C(31)	1.384(6)
C(31)-C(32)	1.376(6)
C(32)-C(33)	1.397(5)
C(34)-C(39)	1.397(5)
C(34)-C(35)	1.406(5)
C(35)-C(36)	1.388(5)
C(36)-C(37)	1.396(5)
C(37)-C(38)	1.367(6)
C(38)-C(39)	1.406(5)
C(40)-C(45)	1.392(4)
C(40)-C(41)	1.405(4)
C(41)-C(42)	1.389(5)
C(42)-C(43)	1.377(5)
C(43)-C(44)	1.381(5)
C(44)-C(45)	1.391(5)
C(46)-C(47)	1.396(5)
C(46)-C(51)	1.399(5)
C(47)-C(48)	1.391(5)
C(48)-C(49)	1.371(6)
C(49)-C(50)	1.379(6)
C(50)-C(51)	1.385(5)
C(52)-C(53)	1.390(5)
C(52)-C(57)	1.408(5)
C(53)-C(54)	1.391(5)
C(54)-C(55)	1.373(6)
C(55)-C(56)	1.366(6)
C(56)-C(57)	1.382(5)
C(58)-C(63)	1.393(5)
C(58)-C(59)	1.403(5)
C(59)-C(60)	1.395(5)
C(60)-C(61)	1.380(6)
C(61)-C(62)	1.370(6)
C(62)-C(63)	1.387(5)
C(64)-C(69)	1.394(4)

C(64)-C(65)	1.403(5)
C(65)-C(66)	1.379(5)
C(66)-C(67)	1.388(5)
C(67)-C(68)	1.380(5)
C(68)-C(69)	1.397(5)
C(70)-C(71)	1.446(6)
N(2)-Co(1)-N(1)	179.30(11)
N(2)-Co(1)-N(3)	93.12(10)
N(1)-Co(1)-N(3)	87.51(10)
N(2)-Co(1)-P(1)	96.88(8)
N(1)-Co(1)-P(1)	83.36(8)
N(3)-Co(1)-P(1)	95.69(7)
N(2)-Co(1)-P(2)	94.38(8)
N(1)-Co(1)-P(2)	85.18(8)
N(3)-Co(1)-P(2)	101.96(7)
P(1)-Co(1)-P(2)	158.48(3)
C(5)-N(1)-C(1)	116.7(2)
C(5)-N(1)-Co(1)	122.8(2)
C(1)-N(1)-Co(1)	120.0(2)
C(6)-P(1)-C(7)	106.56(15)
C(6)-P(1)-C(11)	103.69(15)
C(7)-P(1)-C(11)	112.01(15)
C(6)-P(1)-Co(1)	95.74(10)
C(7)-P(1)-Co(1)	112.04(11)
C(11)-P(1)-Co(1)	123.55(11)
O(1)-B(1)-C(34)	109.4(3)
O(1)-B(1)-C(28)	108.3(3)
C(34)-B(1)-C(28)	117.6(3)
O(1)-B(1)-C(40)	103.1(3)
C(34)-B(1)-C(40)	107.2(3)
C(28)-B(1)-C(40)	110.2(3)
C(3)-O(1)-B(1)	123.3(3)
N(1)-C(1)-C(2)	122.8(3)
N(1)-C(1)-C(6)	116.9(3)
C(2)-C(1)-C(6)	120.2(3)
C(15)-P(2)-C(20)	105.12(15)
C(15)-P(2)-C(16)	103.53(15)
C(20)-P(2)-C(16)	112.90(15)
C(15)-P(2)-Co(1)	98.04(10)
C(20)-P(2)-Co(1)	119.25(11)
C(16)-P(2)-Co(1)	114.80(10)
C(24)-N(2)-Co(1)	173.9(3)
C(58)-B(2)-C(46)	110.9(3)
C(58)-B(2)-C(64)	105.8(3)
C(46)-B(2)-C(64)	111.0(3)
C(58)-B(2)-C(52)	111.6(3)
C(46)-B(2)-C(52)	105.4(3)
C(64)-B(2)-C(52)	112.2(3)
C(1)-C(2)-C(3)	120.6(3)
O(1)-C(3)-C(2)	124.8(3)
O(1)-C(3)-C(4)	118.7(3)
C(2)-C(3)-C(4)	116.4(3)
C(26)-N(3)-Co(1)	167.9(2)
C(5)-C(4)-C(3)	120.9(3)
N(1)-C(5)-C(4)	122.5(3)
N(1)-C(5)-C(15)	115.9(3)
C(4)-C(5)-C(15)	121.3(3)
C(1)-C(6)-P(1)	109.7(2)
C(8)-C(7)-C(9)	108.1(3)
C(8)-C(7)-C(10)	108.3(3)

C(9)-C(7)-C(10)	109.7(3)
C(8)-C(7)-P(1)	109.1(2)
C(9)-C(7)-P(1)	109.5(2)
C(10)-C(7)-P(1)	112.1(2)
C(12)-C(11)-C(14)	108.1(3)
C(12)-C(11)-C(13)	110.0(3)
C(14)-C(11)-C(13)	107.9(3)
C(12)-C(11)-P(1)	111.2(2)
C(14)-C(11)-P(1)	106.8(2)
C(13)-C(11)-P(1)	112.5(2)
C(5)-C(15)-P(2)	110.7(2)
C(17)-C(16)-C(19)	109.5(3)
C(17)-C(16)-C(18)	108.3(3)
C(19)-C(16)-C(18)	107.7(3)
C(17)-C(16)-P(2)	111.3(2)
C(19)-C(16)-P(2)	113.8(2)
C(18)-C(16)-P(2)	105.9(2)
C(23)-C(20)-C(21)	110.8(3)
C(23)-C(20)-C(22)	107.9(3)
C(21)-C(20)-C(22)	109.0(3)
C(23)-C(20)-P(2)	112.7(2)
C(21)-C(20)-P(2)	108.5(2)
C(22)-C(20)-P(2)	107.9(2)
N(2)-C(24)-C(25)	178.1(3)
N(3)-C(26)-C(27)	179.6(4)
C(33)-C(28)-C(29)	115.4(3)
C(33)-C(28)-B(1)	126.7(3)
C(29)-C(28)-B(1)	117.9(3)
C(30)-C(29)-C(28)	123.1(4)
C(29)-C(30)-C(31)	119.7(4)
C(32)-C(31)-C(30)	119.2(4)
C(31)-C(32)-C(33)	120.3(4)
C(28)-C(33)-C(32)	122.2(4)
C(39)-C(34)-C(35)	115.4(3)
C(39)-C(34)-B(1)	122.1(3)
C(35)-C(34)-B(1)	122.0(3)
C(36)-C(35)-C(34)	122.9(3)
C(35)-C(36)-C(37)	119.9(3)
C(38)-C(37)-C(36)	118.8(3)
C(37)-C(38)-C(39)	120.9(4)
C(34)-C(39)-C(38)	122.0(3)
C(45)-C(40)-C(41)	115.9(3)
C(45)-C(40)-B(1)	123.0(3)
C(41)-C(40)-B(1)	121.0(3)
C(42)-C(41)-C(40)	122.6(3)
C(43)-C(42)-C(41)	119.6(3)
C(42)-C(43)-C(44)	119.6(3)
C(43)-C(44)-C(45)	120.3(3)
C(44)-C(45)-C(40)	122.1(3)
C(47)-C(46)-C(51)	114.9(3)
C(47)-C(46)-B(2)	125.1(3)
C(51)-C(46)-B(2)	120.0(3)
C(48)-C(47)-C(46)	122.7(4)
C(49)-C(48)-C(47)	120.4(4)
C(48)-C(49)-C(50)	118.8(4)
C(49)-C(50)-C(51)	120.3(4)
C(50)-C(51)-C(46)	122.8(4)
C(53)-C(52)-C(57)	114.6(3)
C(53)-C(52)-B(2)	119.5(3)
C(57)-C(52)-B(2)	125.8(3)

C(52)-C(53)-C(54)	122.8(3)
C(55)-C(54)-C(53)	120.1(4)
C(56)-C(55)-C(54)	119.3(4)
C(55)-C(56)-C(57)	120.2(4)
C(56)-C(57)-C(52)	122.9(4)
C(63)-C(58)-C(59)	114.6(3)
C(63)-C(58)-B(2)	123.5(3)
C(59)-C(58)-B(2)	121.9(3)
C(60)-C(59)-C(58)	122.9(4)
C(61)-C(60)-C(59)	119.6(4)
C(62)-C(61)-C(60)	119.4(4)
C(61)-C(62)-C(63)	120.1(4)
C(62)-C(63)-C(58)	123.3(4)
C(69)-C(64)-C(65)	115.1(3)
C(69)-C(64)-B(2)	125.4(3)
C(65)-C(64)-B(2)	119.5(3)
C(66)-C(65)-C(64)	123.0(3)
C(65)-C(66)-C(67)	120.3(3)
C(68)-C(67)-C(66)	118.7(3)
C(67)-C(68)-C(69)	120.0(3)
C(64)-C(69)-C(68)	122.9(3)
N(4)-C(71)-C(70)	179.2(5)

Table S 3. Torsion angles [°] for $\mathbf{3}^{\mathbf{BPh_4}}$.

C(34)-B(1)-O(1)-C(3)	86.3(3)
C(28)-B(1)-O(1)-C(3)	-43.0(4)
C(40)-B(1)-O(1)-C(3)	-159.8(3)
C(5)-N(1)-C(1)-C(2)	-1.6(4)
Co(1)-N(1)-C(1)-C(2)	170.5(2)
C(5)-N(1)-C(1)-C(6)	175.6(3)
Co(1)-N(1)-C(1)-C(6)	-12.4(3)
N(1)-C(1)-C(2)-C(3)	2.0(5)
C(6)-C(1)-C(2)-C(3)	-175.1(3)
B(1)-O(1)-C(3)-C(2)	-20.5(5)
B(1)-O(1)-C(3)-C(4)	156.1(3)
C(1)-C(2)-C(3)-O(1)	174.2(3)
C(1)-C(2)-C(3)-C(4)	-2.5(5)
O(1)-C(3)-C(4)-C(5)	-174.0(3)
C(2)-C(3)-C(4)-C(5)	2.9(5)
C(1)-N(1)-C(5)-C(4)	1.9(4)
Co(1)-N(1)-C(5)-C(4)	-169.9(2)
C(1)-N(1)-C(5)-C(15)	-172.2(3)
Co(1)-N(1)-C(5)-C(15)	16.0(4)
C(3)-C(4)-C(5)-N(1)	-2.7(5)
C(3)-C(4)-C(5)-C(15)	171.1(3)
N(1)-C(1)-C(6)-P(1)	-18.4(3)
C(2)-C(1)-C(6)-P(1)	158.8(2)
C(7)-P(1)-C(6)-C(1)	-81.9(2)
C(11)-P(1)-C(6)-C(1)	159.7(2)
Co(1)-P(1)-C(6)-C(1)	33.1(2)
C(6)-P(1)-C(7)-C(8)	52.9(3)
C(11)-P(1)-C(7)-C(8)	165.6(2)
Co(1)-P(1)-C(7)-C(8)	-50.6(3)
C(6)-P(1)-C(7)-C(9)	171.0(2)
C(11)-P(1)-C(7)-C(9)	-76.3(3)
Co(1)-P(1)-C(7)-C(9)	67.5(2)
C(6)-P(1)-C(7)-C(10)	-67.0(3)
C(11)-P(1)-C(7)-C(10)	45.7(3)
Co(1)-P(1)-C(7)-C(10)	-170.5(2)

C(6)-P(1)-C(11)-C(12)	167.5(2)
C(7)-P(1)-C(11)-C(12)	53.0(3)
Co(1)-P(1)-C(11)-C(12)	-86.0(3)
C(6)-P(1)-C(11)-C(14)	-74.7(2)
C(7)-P(1)-C(11)-C(14)	170.8(2)
Co(1)-P(1)-C(11)-C(14)	31.8(3)
C(6)-P(1)-C(11)-C(13)	43.5(3)
C(7)-P(1)-C(11)-C(13)	-71.0(3)
Co(1)-P(1)-C(11)-C(13)	150.0(2)
N(1)-C(5)-C(15)-P(2)	-29.1(3)
C(4)-C(5)-C(15)-P(2)	156.7(2)
C(20)-P(2)-C(15)-C(5)	-97.0(2)
C(16)-P(2)-C(15)-C(5)	144.3(2)
Co(1)-P(2)-C(15)-C(5)	26.3(2)
C(15)-P(2)-C(16)-C(17)	167.0(2)
C(20)-P(2)-C(16)-C(17)	53.9(3)
Co(1)-P(2)-C(16)-C(17)	-87.3(2)
C(15)-P(2)-C(16)-C(19)	42.6(3)
C(20)-P(2)-C(16)-C(19)	-70.5(3)
Co(1)-P(2)-C(16)-C(19)	148.2(2)
C(15)-P(2)-C(16)-C(18)	-75.5(2)
C(20)-P(2)-C(16)-C(18)	171.3(2)
Co(1)-P(2)-C(16)-C(18)	30.1(3)
C(15)-P(2)-C(20)-C(23)	-73.8(3)
C(16)-P(2)-C(20)-C(23)	38.3(3)
Co(1)-P(2)-C(20)-C(23)	177.7(2)
C(15)-P(2)-C(20)-C(21)	163.1(2)
C(16)-P(2)-C(20)-C(21)	-84.8(3)
Co(1)-P(2)-C(20)-C(21)	54.6(3)
C(15)-P(2)-C(20)-C(22)	45.2(3)
C(16)-P(2)-C(20)-C(22)	157.3(2)
Co(1)-P(2)-C(20)-C(22)	-63.4(3)
O(1)-B(1)-C(28)-C(33)	130.3(4)
C(34)-B(1)-C(28)-C(33)	5.7(5)
C(40)-B(1)-C(28)-C(33)	-117.5(4)
O(1)-B(1)-C(28)-C(29)	-48.7(4)
C(34)-B(1)-C(28)-C(29)	-173.3(3)
C(40)-B(1)-C(28)-C(29)	63.5(4)
C(33)-C(28)-C(29)-C(30)	-1.4(5)
B(1)-C(28)-C(29)-C(30)	177.7(3)
C(28)-C(29)-C(30)-C(31)	2.4(6)
C(29)-C(30)-C(31)-C(32)	-0.9(6)
C(30)-C(31)-C(32)-C(33)	-1.5(7)
C(29)-C(28)-C(33)-C(32)	-1.1(6)
B(1)-C(28)-C(33)-C(32)	179.9(4)
C(31)-C(32)-C(33)-C(28)	2.5(7)
O(1)-B(1)-C(34)-C(39)	14.5(4)
C(28)-B(1)-C(34)-C(39)	138.5(3)
C(40)-B(1)-C(34)-C(39)	-96.7(3)
O(1)-B(1)-C(34)-C(35)	-174.3(3)
C(28)-B(1)-C(34)-C(35)	-50.2(4)
C(40)-B(1)-C(34)-C(35)	74.5(4)
C(39)-C(34)-C(35)-C(36)	-0.8(5)
B(1)-C(34)-C(35)-C(36)	-172.6(3)
C(34)-C(35)-C(36)-C(37)	1.7(5)
C(35)-C(36)-C(37)-C(38)	-1.1(5)
C(36)-C(37)-C(38)-C(39)	-0.3(6)
C(35)-C(34)-C(39)-C(38)	-0.6(5)
B(1)-C(34)-C(39)-C(38)	171.1(3)
C(37)-C(38)-C(39)-C(34)	1.2(6)

O(1)-B(1)-C(40)-C(45)	-18.2(4)
C(34)-B(1)-C(40)-C(45)	97.3(3)
C(28)-B(1)-C(40)-C(45)	-133.6(3)
O(1)-B(1)-C(40)-C(41)	165.3(3)
C(34)-B(1)-C(40)-C(41)	-79.2(4)
C(28)-B(1)-C(40)-C(41)	49.9(4)
C(45)-C(40)-C(41)-C(42)	-2.2(5)
B(1)-C(40)-C(41)-C(42)	174.6(3)
C(40)-C(41)-C(42)-C(43)	1.3(5)
C(41)-C(42)-C(43)-C(44)	0.1(5)
C(42)-C(43)-C(44)-C(45)	-0.6(5)
C(43)-C(44)-C(45)-C(40)	-0.4(5)
C(41)-C(40)-C(45)-C(44)	1.7(5)
B(1)-C(40)-C(45)-C(44)	-174.9(3)
C(58)-B(2)-C(46)-C(47)	124.2(3)
C(64)-B(2)-C(46)-C(47)	6.9(4)
C(52)-B(2)-C(46)-C(47)	-114.9(3)
C(58)-B(2)-C(46)-C(51)	-57.7(4)
C(64)-B(2)-C(46)-C(51)	-175.1(3)
C(52)-B(2)-C(46)-C(51)	63.2(4)
C(51)-C(46)-C(47)-C(48)	0.7(5)
B(2)-C(46)-C(47)-C(48)	178.9(3)
C(46)-C(47)-C(48)-C(49)	0.1(5)
C(47)-C(48)-C(49)-C(50)	-0.7(5)
C(48)-C(49)-C(50)-C(51)	0.3(5)
C(49)-C(50)-C(51)-C(46)	0.5(5)
C(47)-C(46)-C(51)-C(50)	-1.0(5)
B(2)-C(46)-C(51)-C(50)	-179.3(3)
C(58)-B(2)-C(52)-C(53)	-178.4(3)
C(46)-B(2)-C(52)-C(53)	61.1(4)
C(64)-B(2)-C(52)-C(53)	-59.9(4)
C(58)-B(2)-C(52)-C(57)	6.7(5)
C(46)-B(2)-C(52)-C(57)	-113.8(4)
C(64)-B(2)-C(52)-C(57)	125.2(3)
C(57)-C(52)-C(53)-C(54)	-0.4(5)
B(2)-C(52)-C(53)-C(54)	-175.9(3)
C(52)-C(53)-C(54)-C(55)	0.0(5)
C(53)-C(54)-C(55)-C(56)	0.7(5)
C(54)-C(55)-C(56)-C(57)	-1.0(6)
C(55)-C(56)-C(57)-C(52)	0.6(6)
C(53)-C(52)-C(57)-C(56)	0.1(5)
B(2)-C(52)-C(57)-C(56)	175.3(3)
C(46)-B(2)-C(58)-C(63)	-16.4(5)
C(64)-B(2)-C(58)-C(63)	104.1(4)
C(52)-B(2)-C(58)-C(63)	-133.6(3)
C(46)-B(2)-C(58)-C(59)	166.4(3)
C(64)-B(2)-C(58)-C(59)	-73.0(4)
C(52)-B(2)-C(58)-C(59)	49.3(4)
C(63)-C(58)-C(59)-C(60)	1.5(5)
B(2)-C(58)-C(59)-C(60)	178.9(4)
C(58)-C(59)-C(60)-C(61)	-1.5(6)
C(59)-C(60)-C(61)-C(62)	-0.5(6)
C(60)-C(61)-C(62)-C(63)	2.4(6)
C(61)-C(62)-C(63)-C(58)	-2.4(6)
C(59)-C(58)-C(63)-C(62)	0.4(5)
B(2)-C(58)-C(63)-C(62)	-176.9(3)
C(58)-B(2)-C(64)-C(69)	116.8(3)
C(46)-B(2)-C(64)-C(69)	-122.7(3)
C(52)-B(2)-C(64)-C(69)	-5.1(4)
C(58)-B(2)-C(64)-C(65)	-61.0(4)

C(46)-B(2)-C(64)-C(65)	59.5(4)
C(52)-B(2)-C(64)-C(65)	177.1(3)
C(69)-C(64)-C(65)-C(66)	0.5(5)
B(2)-C(64)-C(65)-C(66)	178.5(3)
C(64)-C(65)-C(66)-C(67)	-1.5(5)
C(65)-C(66)-C(67)-C(68)	1.3(5)
C(66)-C(67)-C(68)-C(69)	-0.2(5)
C(65)-C(64)-C(69)-C(68)	0.5(4)
B(2)-C(64)-C(69)-C(68)	-177.3(3)
C(67)-C(68)-C(69)-C(64)	-0.7(5)

Analytical Data

UV/Vis and UV/Vis SEC Data

UV/Vis Titration Data

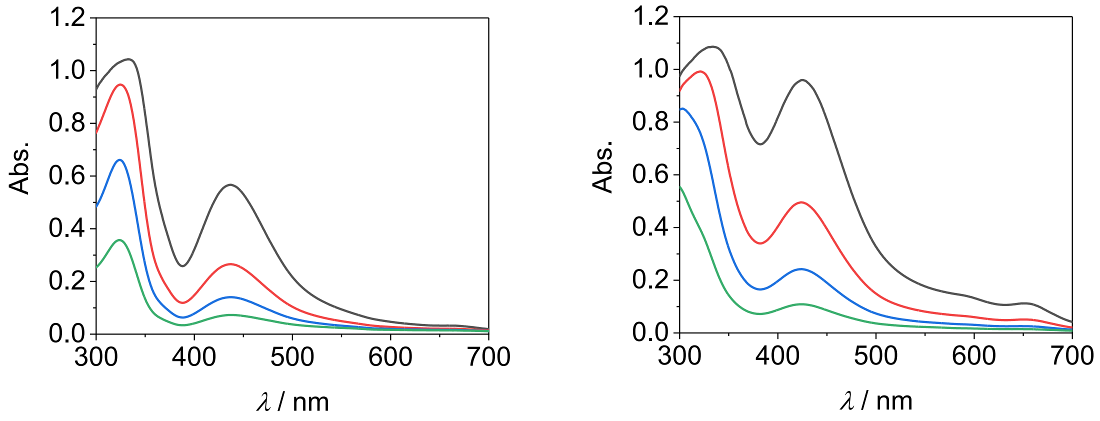

*Figure S 2. Concentration dependent data of **2H**$^{2+}$, c = 0.4, 0.2, 0.1, 0.05 mM (left) and **2**$^{+}$, c = 0.4, 0.2, 0.1, 0.05 mM (right) in MeCN.*

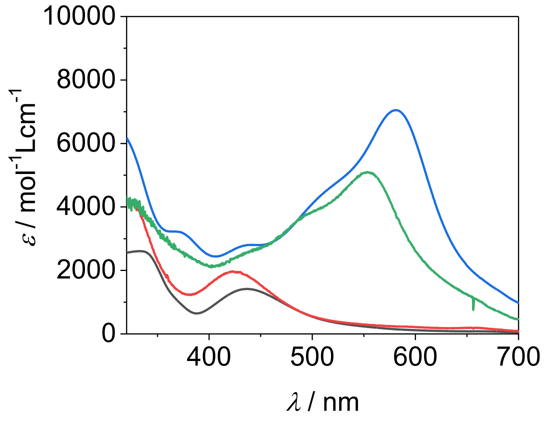

*Figure S 3. Overlay of the UV/Vis data of **2**$^{+}$ (red), **2H**$^{2+}$ (black), **2** (green), and **2H**$^{+}$ (blue) in MeCN for comparison.*

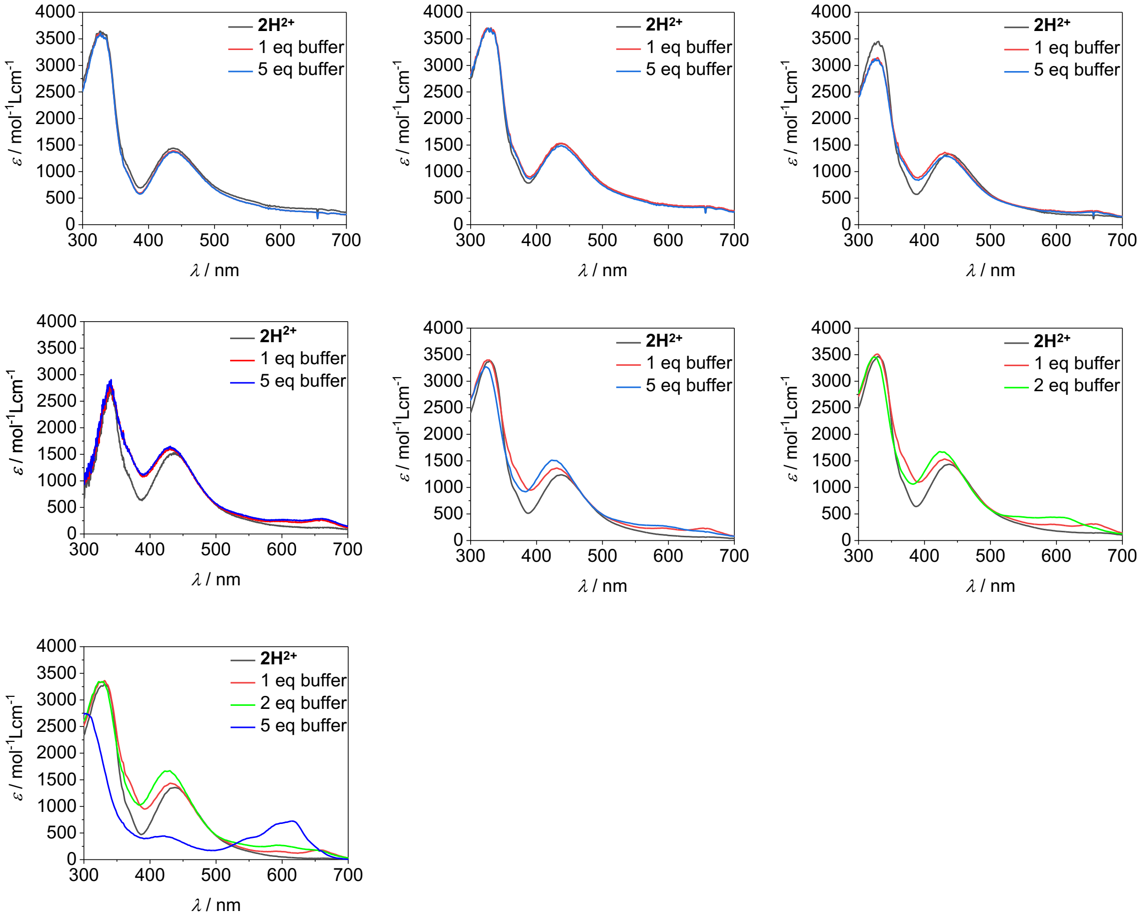

*Figure S 4: UV/Vis titration of **2H**$^{2+}$ (c ~ 0.25 mM) with different X/XH buffer system in MeCN; where X = benzosulphonamide (first row, left), 2,4,6- trimethylpyridine (first row, middle), benzylamine (first row, right), triethylamine (second row, left), 1,1,3,3-tetramethylguanidin (second row, middle), diazabicycloundecene (second row, right), triazabicyclodecene (third row).*

Table S 4: pK_a values of different XH/X buffer systems.

X	pK_a
benzosulphonamide	14.5
2,4,6- trimethylpyridine	15.0
benzylamine	16.9
triethylamine	18.8
1,1,3,3-tetramethylguanidin	23.3
diazabicycloundecene	24.3
Triazabicyclodecene	26.0

(Spectro)electrochemical data

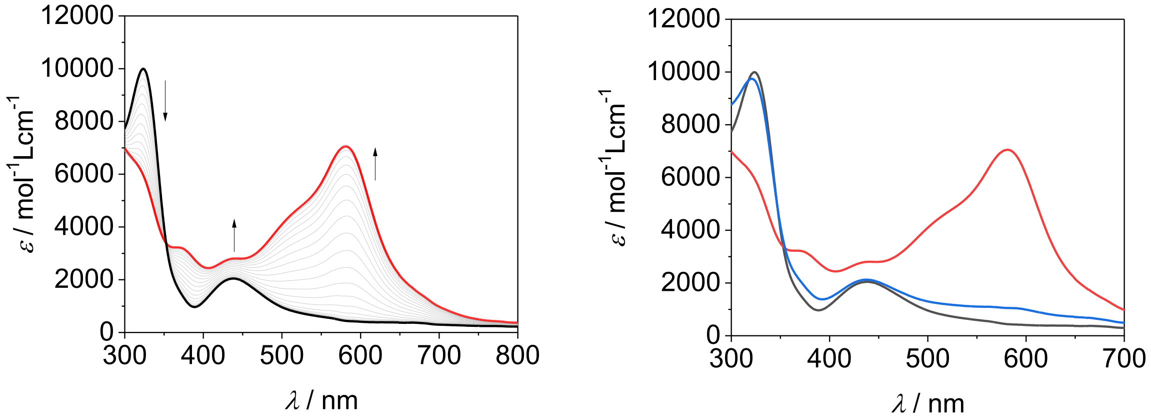

Figure S 5. Left: UV/Vis SEC reduction of ***2H***$^{2+}$*(black) forming* ***2H***$^{+}$*(red) in MeCN; Right: after reoxidation (blue) in MeCN, c ~ 0.75 mM, I = 0.1 M nBu*$_4$*NPF*$_6$*.*

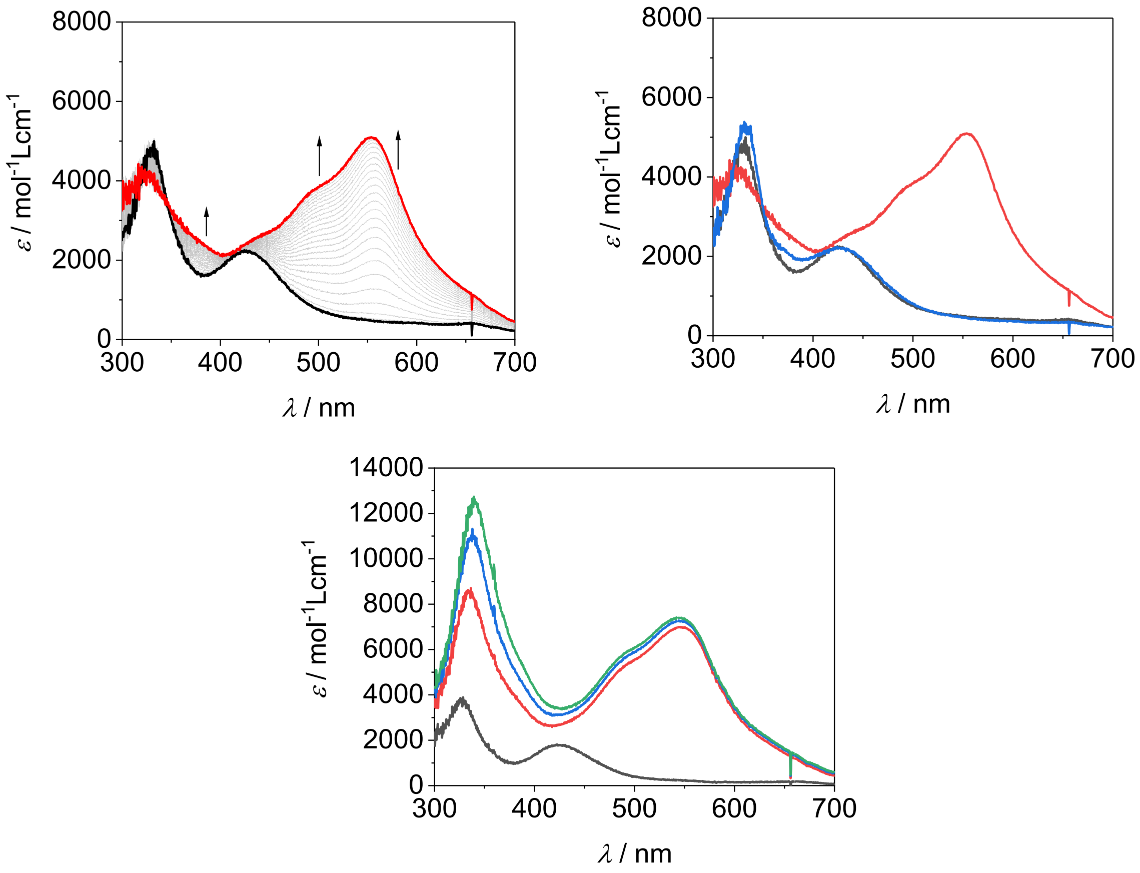

Figure S 6. Top: UV/Vis SEC of **2**$^{+}$*(black) in MeCN after reduction (red) after reoxidation (blue); Bottom: Redox titration of* **2**$^{+}$ *with 0 (black) 1 (red), 2 (blue), and 3 (green) equiv. of Cp*$_2$*Co.*

1e$^-$/1H$^+$-Reactivity of **2H**$^+$

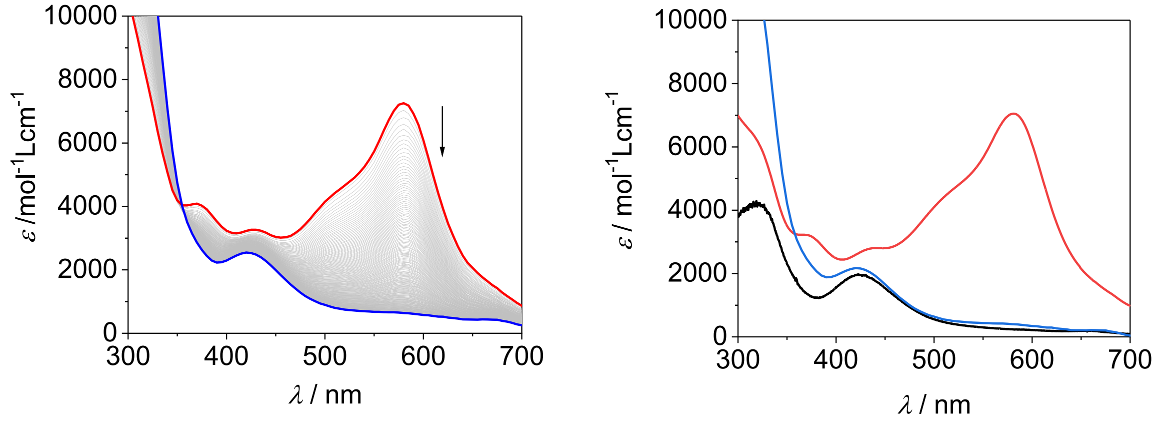

Figure S 7. Left: Change of UV/Vis spectrum of ***2H***$^+$ *(red) over time (blue), c ~ 0.075 mM; Right: Comparison of UV/Vis spectra of* ***2H***$^+$ *over time (blue) with* ***2***$^+$ *(black).*

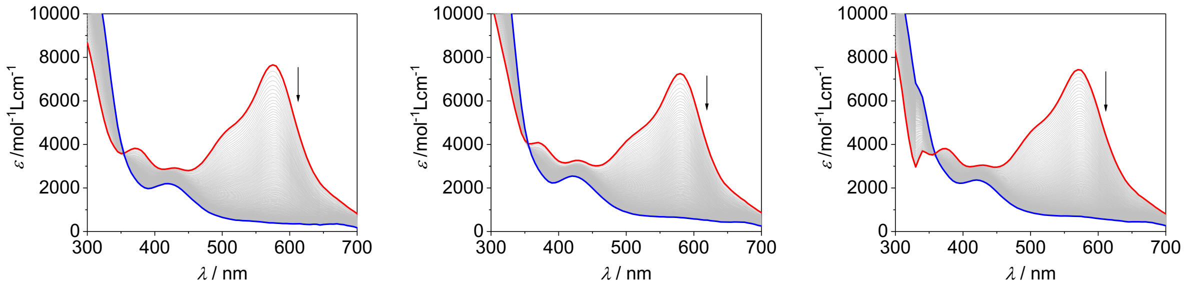

Figure S 8. UV/Vis spectra of ***2H***$^+$ *over time at different concentration: 0.06 mM (left), 0.075 mM (middle), 0.125 mM (right).*

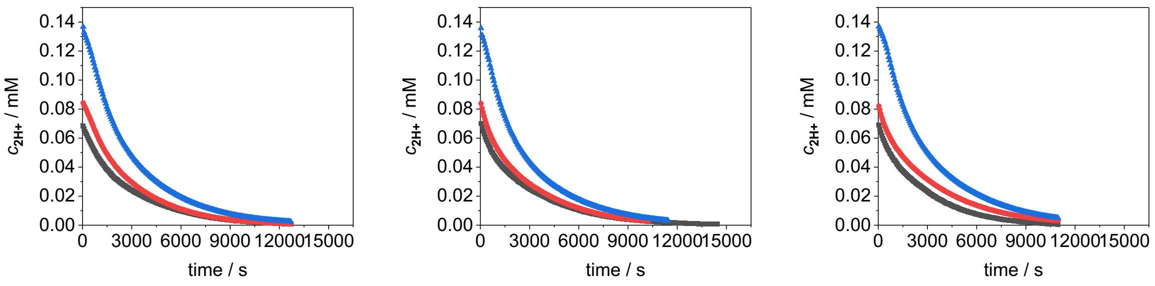

Figure S 9. Change of the concentration of ***2H***$^+$ *over time, different initial concentrations, 0.06 mM (black), 0.075 mM (red), 0.125 mM (blue), 298 K; three independent runs.*

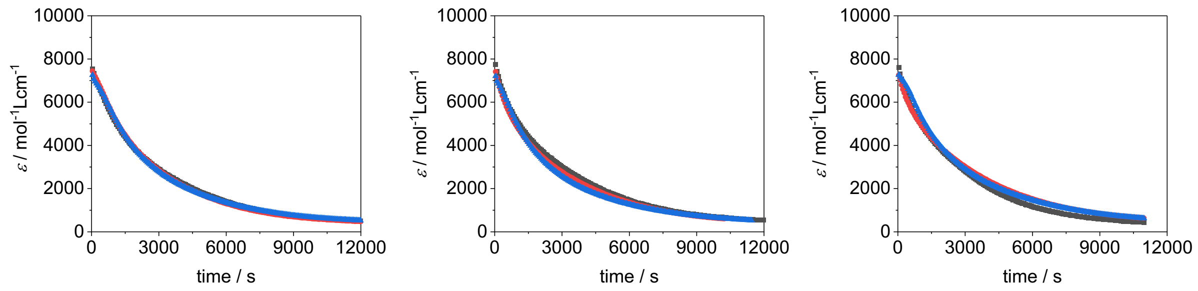

Figure S 10. Change of the extinction at 581 nm of ***2H***$^{+}$ *over time, different initial concentrations, 0.06 mM (black), 0.075 mM (red), 0.125 mM (blue), 298 K; three independent runs.*

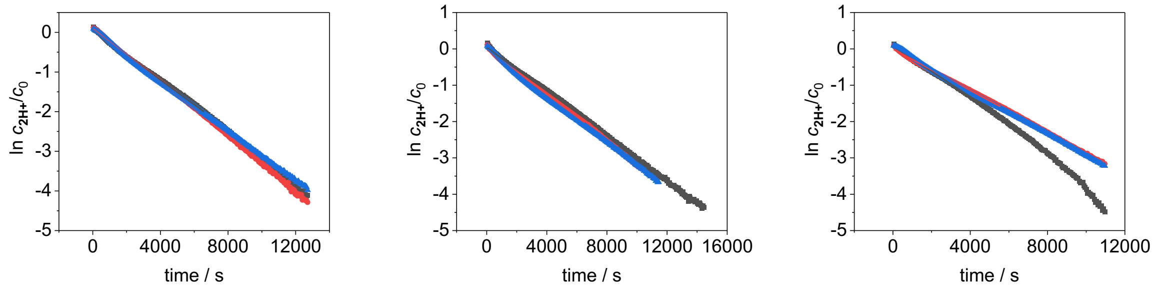

Figure S 11. First order linearization plots of the normalized concentration of ***2H***$^{+}$ *over time, different initial concentration, 0.06 mM (black), 0.075 mM (red), 0.125 mM (blue), 298 K; three independent runs.*

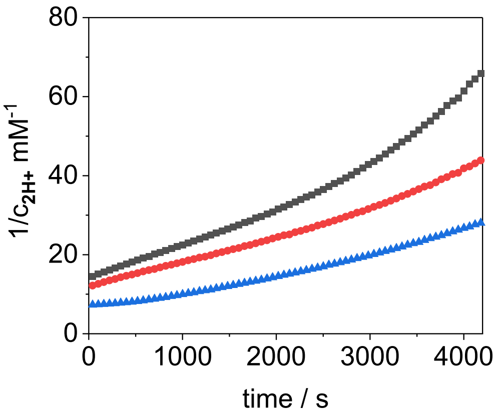

Figure S 12. Representative second order linearization plots of the decay experiment of ***2H***$^{+}$*, different initial concentration, 0.06 mM (black), 0.075 mM (red), 0.125 mM (blue), 298 K.*

*Table S 5. Slope and rate constants /s^{-1} for the decay of **2H$^+$** at different concentrations, 298 K.*

Conc./ mM	Slope Run 1	Slope Run 2	Slope Run 3	Rate constant	Standard deviation
0.06	3.11×10^{-4}	2.99×10^{-4}	3.49×10^{-4}	3.2×10^{-4}	0.3×10^{-4}
0.075	3.35×10^{-4}	3.08×10^{-4}	2.84×10^{-4}	3.1×10^{-4}	0.3×10^{-4}
0.125	3.28×10^{-4}	3.37×10^{-4}	3.19×10^{-5}	1.3×10^{-4}	0.1×10^{-4}

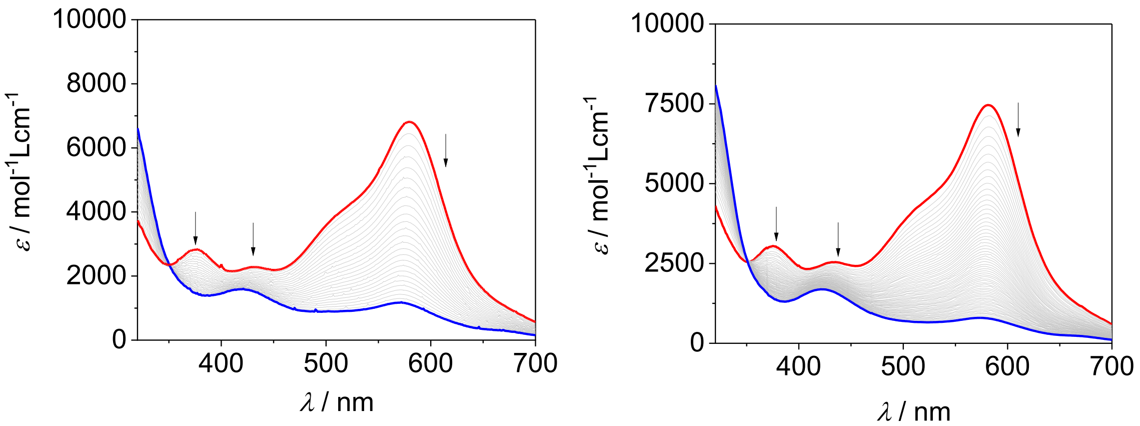

*Figure S 13. Representative UV/Vis spectra of **2H$^+$** in MeCN over time at 293 K (left) and 288 K (right), c ~ 0.075 mM.*

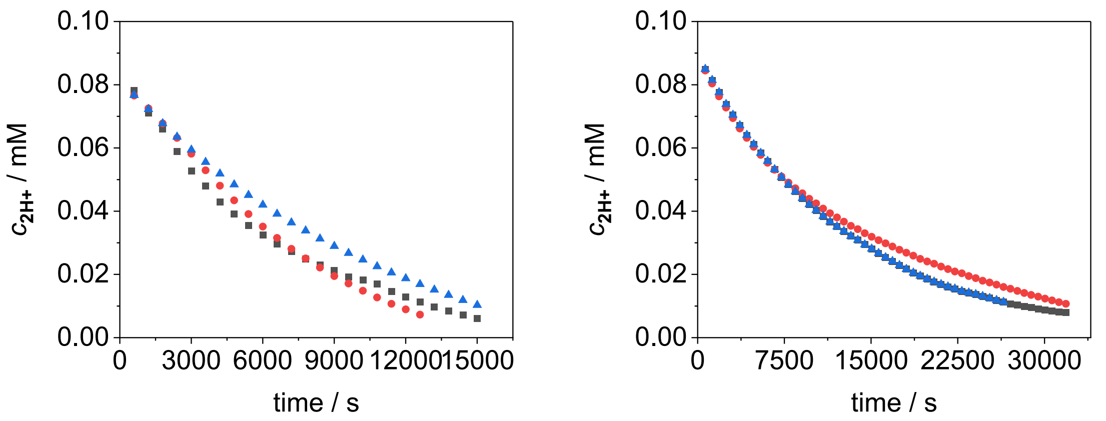

*Figure S 14. Change of the concentration of **2H$^+$** over time, different temperatures, 293 K (left), 288 K (right), 0.075 mM; three independent runs.*

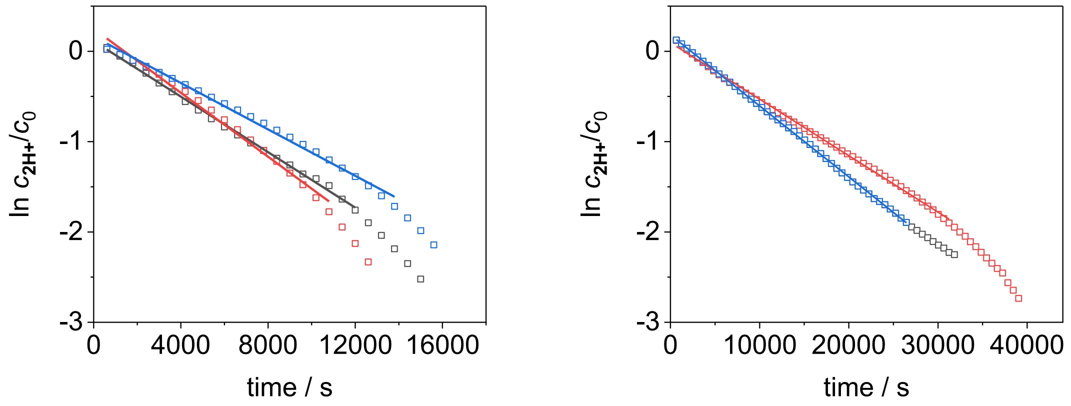

Figure S 15. First order linearization plots of the normalized concentration of ***2H***$^{+}$ *over time, 293 K (left), 288 K (right), 0.075 mM; three independent runs.*

Table S 6. Slope and rate constants /s^{-1} *for the decay of* ***2H***$^{+}$ *at different temperatures, 0.075 mM.*

Temp.	Slope			Rate constant	Standard deviation
	Run 1	Run 2	Run 3		
298	2.84×10^{-4}	3.08×10^{-4}	3.35×10^{-4}	3.1×10^{-4}	0.3×10^{-4}
293	1.54×10^{-4}	1.77×10^{-4}	1.28×10^{-4}	1.5×10^{-4}	0.2×10^{-4}
288	0.79×10^{-4}	0.63×10^{-4}	0.79×10^{-4}	0.73×10^{-4}	0.09×10^{-4}

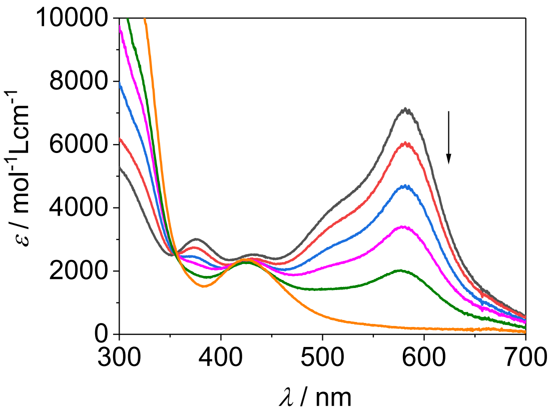

Figure S 16: Reaction of ***2H***$^{+}$ *(0.075mM) with 0.2, 0.4, 0.6, 0.8, 1.0 equiv. of 2,4,6- tri-tert-butyl phenoxy radical in MeCN at rt.*

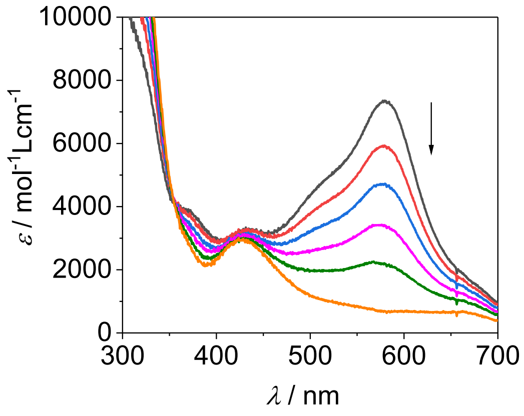

*Figure S 17: Reaction of **2H**$^+$ (0.075 mM) with 0.2, 0.4, 0.6, 0.8, 1.0 equiv. of TEMPO in MeCN at rt.*

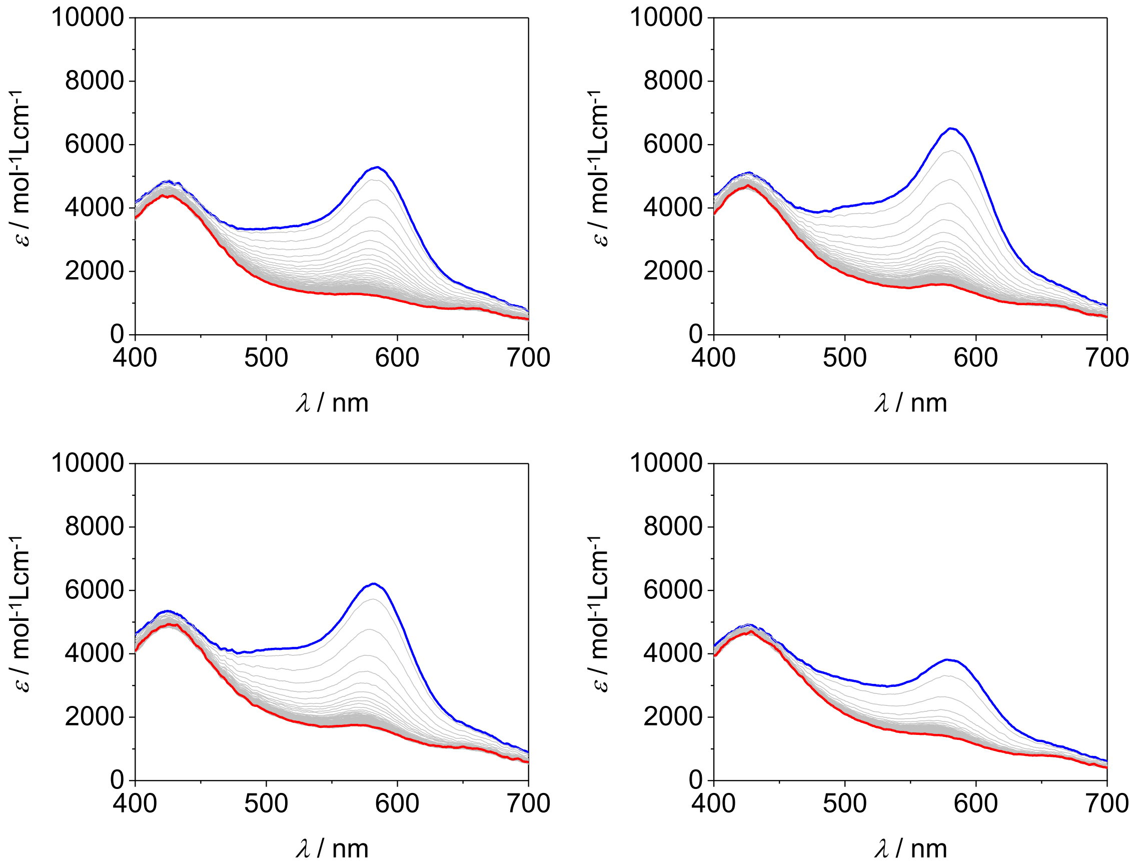

*Figure S 18. Representative UV/Vis spectra of **2H**$^+$ and 10 (top left), 15 (top right), 20 (bottom left), and 30 (bottom right) equiv. of TEMPO in MeCN over time at −35 °C; blue: initial kinetic trace after mixing, red: last kinetic trace.*

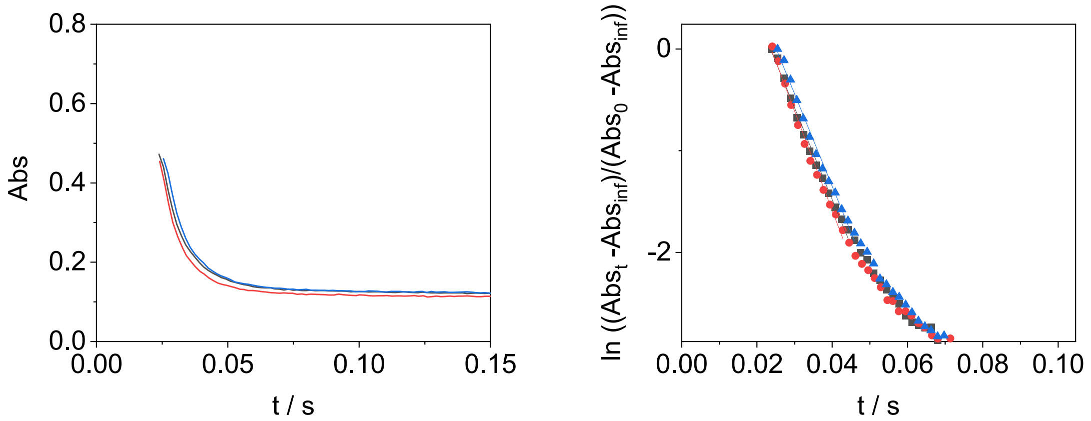

*Figure S 19. Absorption (left) and linearization plots (right) of **2H**$^+$ and 10 equiv. of TEMPO in MeCN over time at −35 °C; three independent runs.*

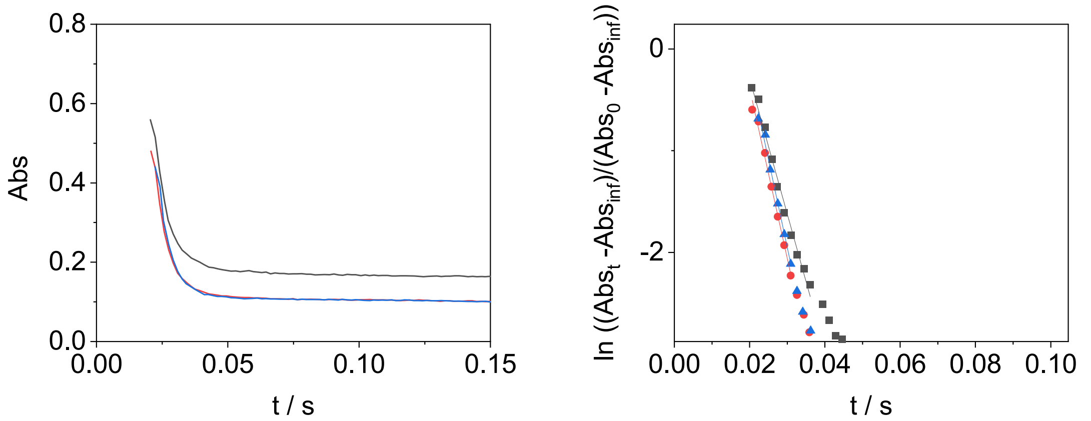

*Figure S 20. Absorption (left) and linearization plots (right) of **2H**$^+$ and 15 equiv. of TEMPO in MeCN over time at −35 °C; three independent runs.*

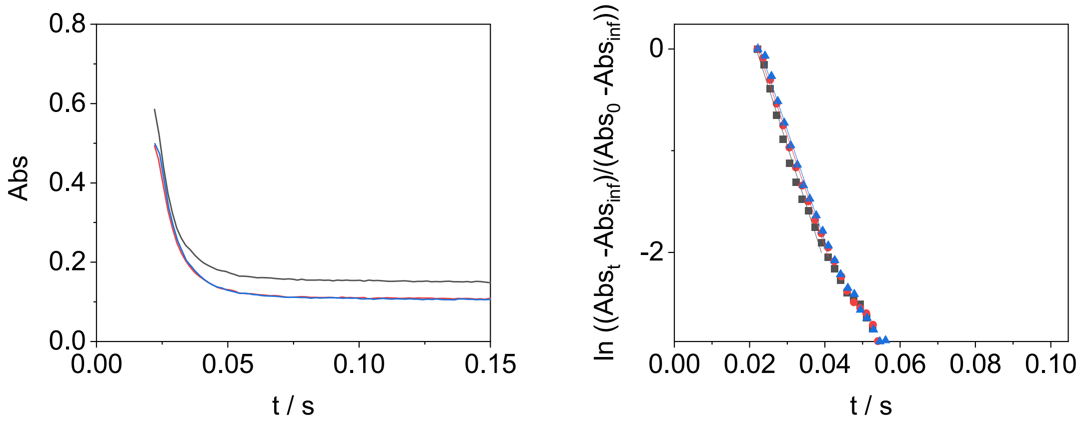

*Figure S 21. Absorption (left) and linearization plots (right) of **2H**$^+$ and 20 equiv. of TEMPO in MeCN over time at −35 °C; three independent runs.*

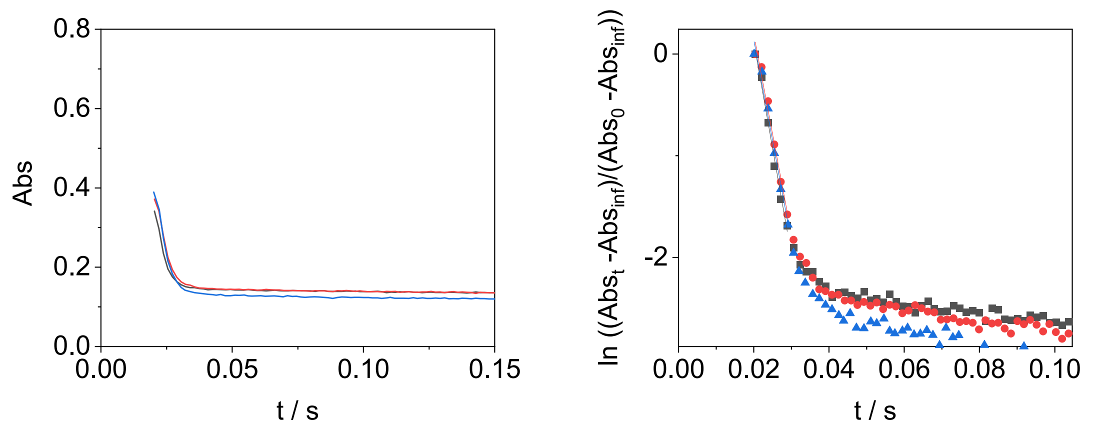

*Figure S 22. Absorption (left) and linearization plots (right) of **2H**$^+$ and 30 equiv. of TEMPO in MeCN over time at −35 °C; three independent runs.*

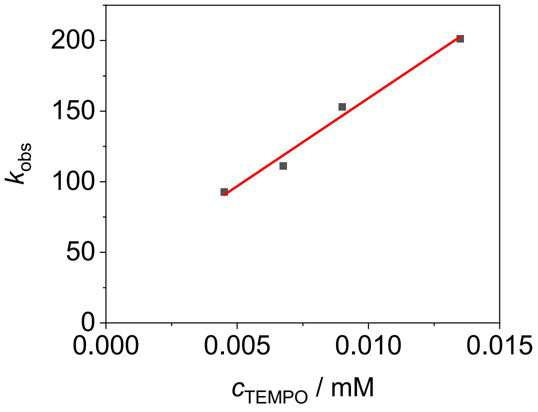

Figure S 23. k_{obs} vs. concentration of TEMPO$^•$, MeCN, −35 °C.

*Table S 7. Pseudo first order rate constants /s^{-1} for the reaction of **2H**$^+$ with TEMPO$^•$, −35 °C.*

Conc. TEMPO/	Slope			Rate constant	Standard deviation
mM	Run 1	Run 2	Run 3		
4.5	9.0×10^1	9.8×10^1	9.1×10^1	0.93×10^2	0.03×10^2
6.75	1.2×10^2	1.1×10^2	1.1×10^2	1.1×10^2	0.3×10^2
9.0	1.3×10^2	1.6×10^2	1.7×10^2	1.5×10^2	0.2×10^2
13.5	2.1×10^2	2.0×10^2	2.0×10^2	2.0×10^2	0.1×10^2

Electrochemical Data

CV Data

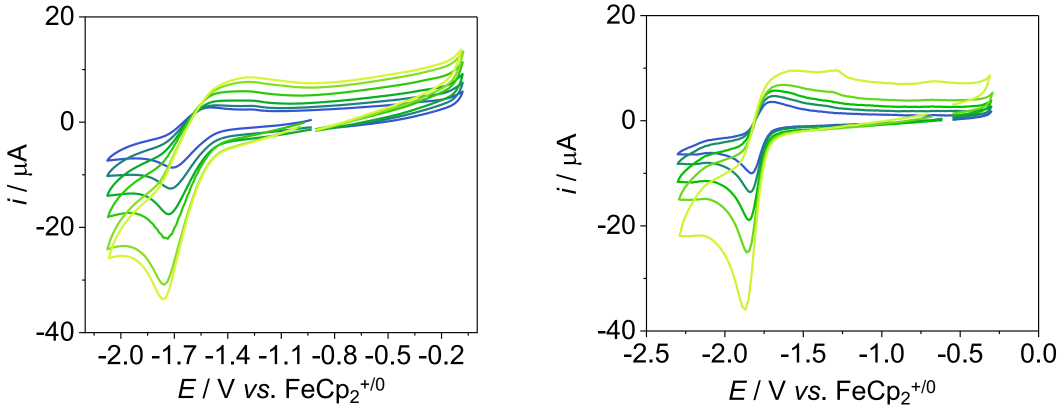

*Figure S 24. Scan rate dependent CV data of **1** in DMF, c ~ 1mM; left: I = 0.1 M nBu$_4$NPF$_6$, v = 0.05, 0.1, 0.2, 0.4, 0.8 1.0 Vs^{-1}; right: I = 0.1 M nBu$_4$NCl, v = 0.05, 0.1, 0.2, 0.5, 1.0 Vs^{-1}(right).*

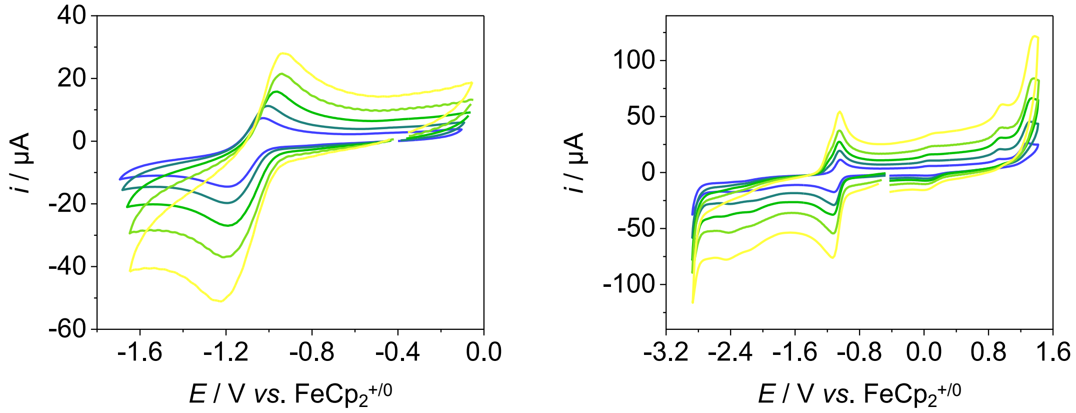

*Figure S 25. Scan rate dependent CV data of **2H**$^{2+}$ in MeCN, c ~ 1mM, I = 0.1 M nBu$_4$NPF$_6$, v = 0.05, 0.1, 0.2, 0.5, 1.0 Vs^{-1}.*

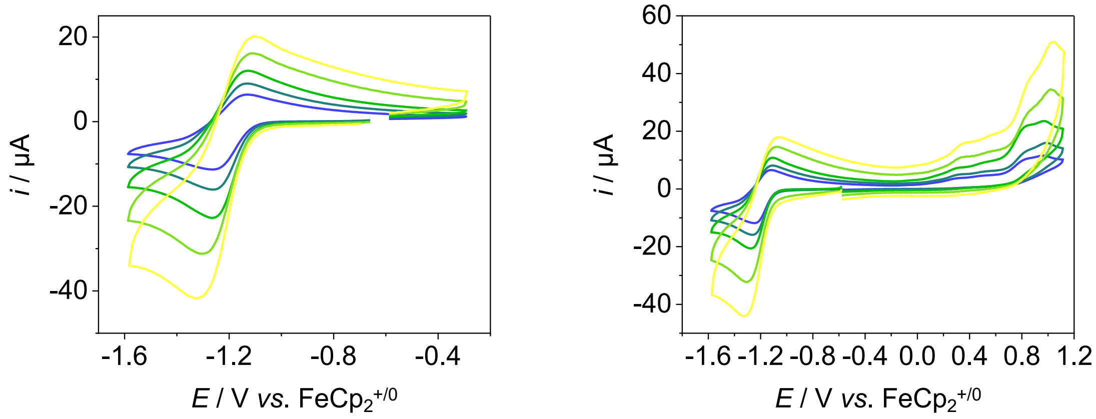

Figure S 26: Scan rate dependent CV data of $\mathbf{2}^{+}$*(right) in MeCN, c ~ 1mM, I = 0.1 M nBu*$_4$*NPF*$_6$*, v = 0.05, 0.1, 0.2, 0.5, 1.0 Vs*$^{-1}$*.*

EPR Data

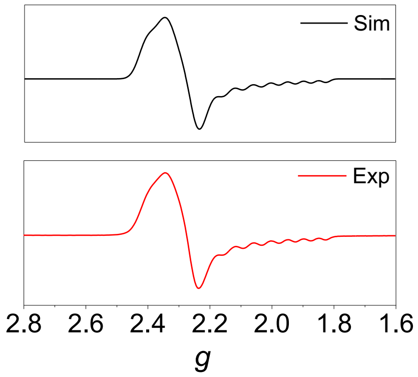

Figure S 27. EPR spectra of $\mathbf{2H}^{2+}$ *in MeCN at 147K; simulation parameter:* $g_x = 2.00$, $g_y = 2.28$, $g_z = 2.33$, A_x *(1 ×* ^{59}*Co) = 263 MHz,* A_y *(1 ×* ^{59}*Co) = 20 MHz, and* A_z *(1 ×* ^{59}*Co) = 85 MHz.*

IR Data

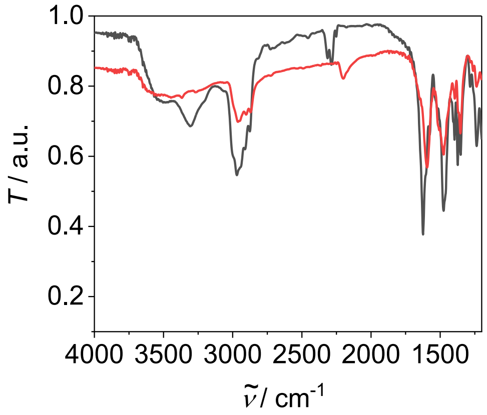

Figure S 28. KBr-IR spectra of $\mathbf{2H}^{2+}$ *(black) and* $\mathbf{2}^{+}$ *(red).*

GC-WLD Data

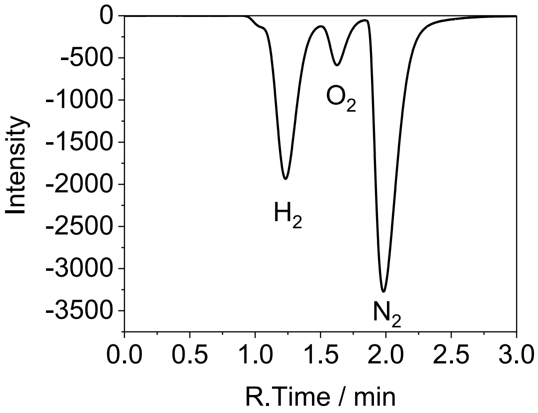

*Figure S 29. GC-TCD trace of the headspace after the reaction of **2H**$^{2+}$ and cobaltocene forming **2**$^{+}$.*

NMR Data

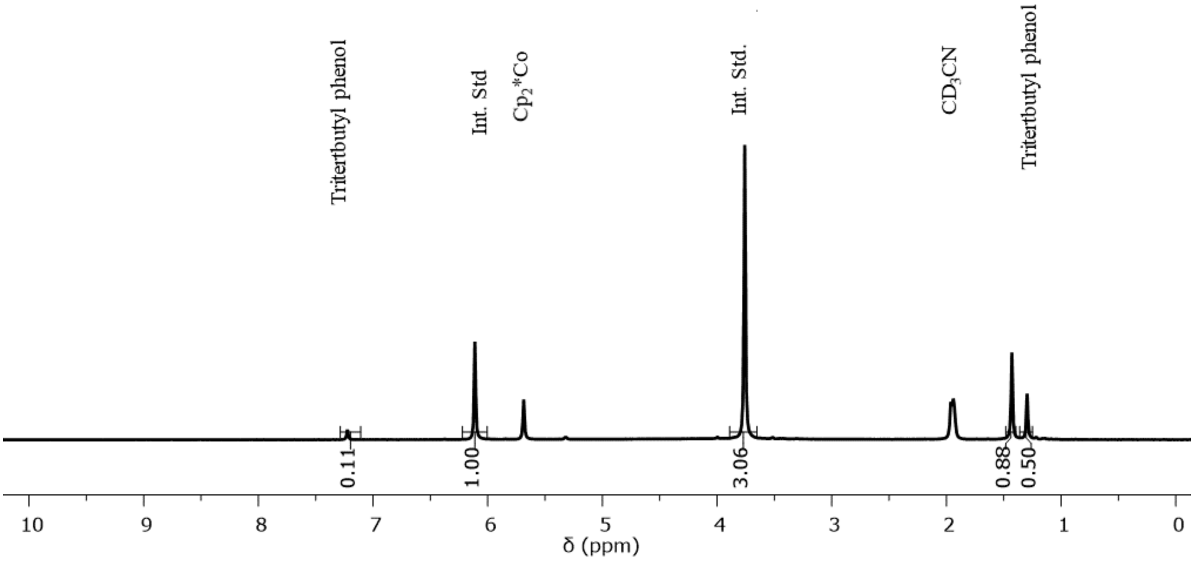

*Figure S 30. ^{1}H NMR of **2H**$^{+}$ with 2,4,6-tri-tert-butylphenoxy radical in CD$_3$CN in presence of trimethoxybenzene as internal. standard.*

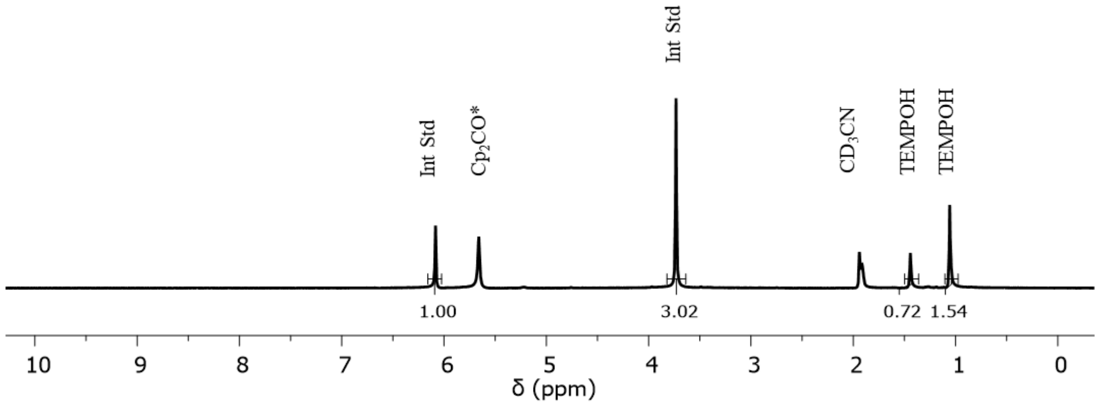

*Figure S 31: ^{1}H NMR of **2H**+ with TEMPO• in CD3CN in presence of trimethoxybenzene as internal standard.*

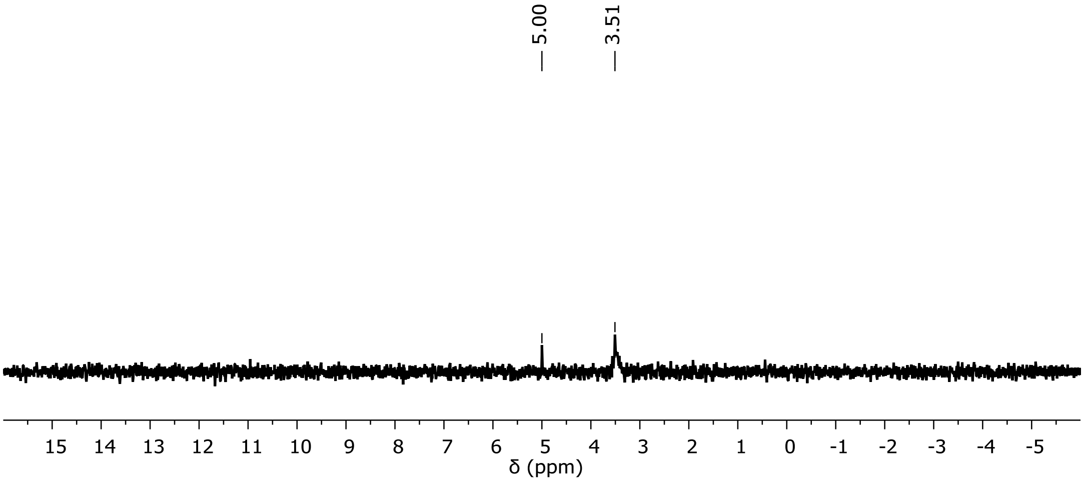

*Figure S 32. ^{2}H NMR spectra of **2D**+ over time in CH3CN.*

Computational Section

TD-DFT Data

UV/Vis Spectra

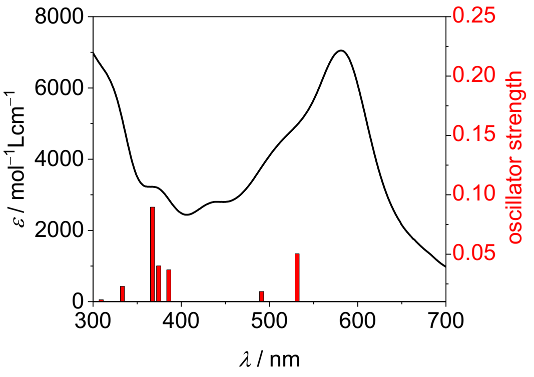

Figure S 33. Comparison of vertical excited energies (red bars) with experimentally obtained UV/vis spectrum of ***2H****$^{+}$ (black trace) in MeCN. TD-DFT: SCS-wPBEPP86/def2-TZVP @ CPCM(MeCN)*

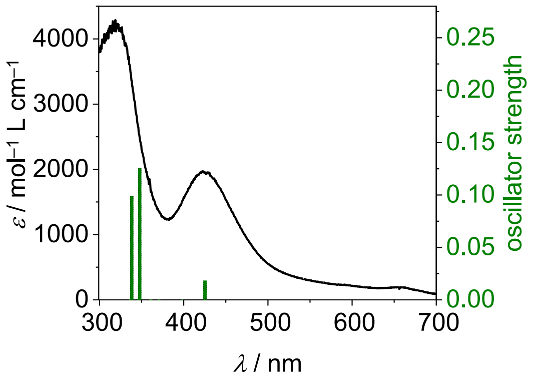

Figure S 34. Comparison of vertical excited energies (green bars) with experimentally obtained UV/vis spectrum of ***2****$^{+}$ (black trace) in MeCN. TD-DFT: SCS-wPBEPP86/def2-TZVP @ CPCM(MeCN)*

Results of the BDFE Calculations

Table S 8. BDFE of ***2H****$^{+}$ at 298 K with different density functionals and basis sets.*

Density functional and basis set	ΔG^{0}_{298K} of **2H**$^{+}$ (kcal/mol)
TPSSh/def2-TZVP	55.8
B3LYP/def2-TZVP	50.9
M06/def2-TZVP	54.8

Potential transformation of **2H**$^+$ to **2'H**$^+$

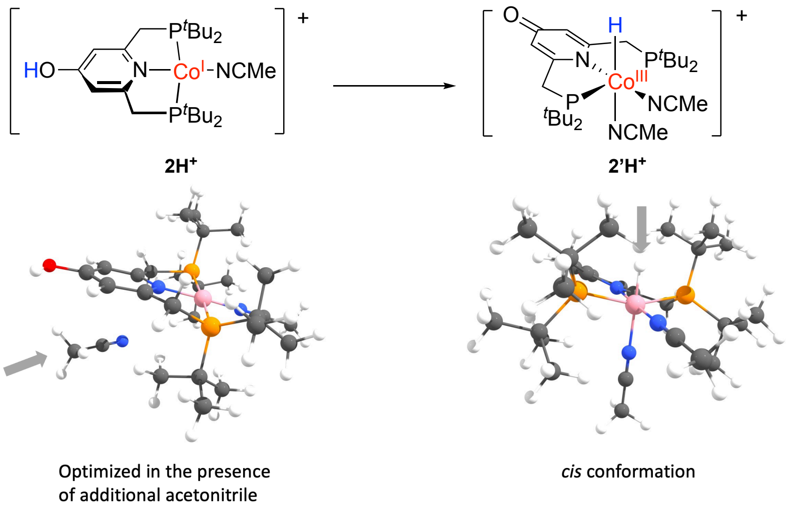

*Figure S 35. Reaction scheme and optimized structures of the **2H**$^+$ to **2'H**$^+$ transformation, where **2H**$^+$ is optimized in the presence of an additional acetonitrile solvent molecule to account for the 6-fold Co-coordination of **2'H**$^+$. A cis conformation of **2'H**$^+$ is more stable by 21.7 kcal/mol than trans.*

References

[1] (a) Hathaway, B. J.; Holah, D. G.; Underhill, A. E. The preparation and properties of some bivalent transition-metal tetrafluoroborate–methyl cyanide complexes, *J. Chem. Soc.* **1962**, 2444−2448, DOI: 10.1039/JR9620002444; (b) Müller, J.; Scheer, M. Coordination Behavior of a P4-Butterfly Complex towards Transition Metal Lewis Acids: Preservation versus Rearrangement. *Chem. Eur. J.* **2021,** *27* (11), 3675–3681. DOI: 10.1002/chem.202005025.

[2] Mukherjee, J.; Ostermann, N.; Aniban, X.; Safianova, I.; Rotthowe, N.; Mata, R.; Siewert, I. A Nickel Complex with a Proton-Responsive PNP Pincer-Type Ligand as a Proton-Coupled Electron Transfer Reagent. *Organometallics* **2023,** *42*, 3258−3265. DOI: 10.1021/acs.organomet.3c00378

[3] Thomas, A. M.; Lin, B.-L.; Wasinger, E. C.; Stack, T. D. P. Ligand noninnocence of thiolate/disulfide in dinuclear copper complexes: solvent-dependent redox isomerization and proton-coupled electron transfer. *J. Am. Chem. Soc.* **2013,** *135*, 18912−18919. DOI: 10.1021/ja409603m

[4] (a) APEX3 v2016.9-0 (SAINT/SADABS/SHELXT/SHELXL), Bruker AXS Inc., Madison, WI, USA, **2016**; (b) Sheldrick. G. M. SHELXT – Integrated space-group and crystal structure determination. *Acta Cryst.* **2015**, A71, 3-8. DOI:10.1107/S2053273314026370; (c) Sheldrick. G. M. Crystal structure refinement with SHELXL. *Acta Cryst.*, **2015**, C71, 3-8. DOI: 10.1107/S2053229614024218; (d) Sheldrick. G. M. A short history of SHELX. *Acta Cryst.*, **2008**, A64, 112-122. DOI: 10.1107/S0108767307043930.
